# Large-scale analysis of *DFNA5* methylation reveals its potential as biomarker for breast cancer

**DOI:** 10.1186/s13148-018-0479-y

**Published:** 2018-04-11

**Authors:** Lieselot Croes, Matthias Beyens, Erik Fransen, Joe Ibrahim, Wim Vanden Berghe, Arvid Suls, Marc Peeters, Patrick Pauwels, Guy Van Camp, Ken Op de Beeck

**Affiliations:** 1 Center of Medical Genetics, University of Antwerp and Antwerp University Hospital, Prins Boudewijnlaan 43/6, BE-2650 Edegem, Antwerp Belgium; 2 Center for Oncological Research, University of Antwerp and Antwerp University Hospital, Universiteitsplein 1, BE-2610 Wilrijk, Antwerp Belgium; 3 StatUa Center for Statistics, University of Antwerp, Prinsstraat 13, BE-2000 Antwerp, Belgium; 4 Laboratory of Protein Chemistry, Proteomics and Epigenetic Signaling (PPES), University of Antwerp, Universiteitsplein 1, BE-2610 Wilrijk, Antwerp Belgium

**Keywords:** DFNA5, Methylation, Breast cancer, Detection biomarker, Prognostic biomarker, TCGA

## Abstract

**Background:**

Breast cancer is the most frequent cancer among women worldwide. Biomarkers for early detection and prognosis of these patients are needed. We hypothesized that *deafness*, *autosomal dominant 5* (*DFNA5*) may be a valuable biomarker, based upon strong indications for its role as tumor suppressor gene and its function in regulated cell death. In this study, we aimed to analyze *DFNA5* methylation and expression in the largest breast cancer cohort to date using publicly available data from TCGA, in order to further unravel the role of *DFNA5* as detection and/or prognostic marker in breast cancer. We analyzed Infinium HumanMethylation450k data, covering 22 different CpGs in the *DFNA5* gene (668 breast adenocarcinomas and 85 normal breast samples) and *DFNA5* expression (Agilent 244K Custom Gene Expression: 476 breast adenocarcinomas and 56 normal breast samples; RNA-sequencing: 666 breast adenocarcinomas and 71 normal breast samples).

**Results:**

*DFNA5* methylation and expression were significantly different between breast cancer and normal breast samples. Overall, breast cancer samples showed higher *DFNA5* methylation in the putative gene promoter compared to normal breast samples, whereas in the gene body and upstream of the putative gene promoter, the opposite is true. Furthermore, *DFNA5* methylation, in 10 out of 22 CpGs, and expression were significantly higher in lobular compared to ductal breast cancers. An important result of this study was the identification of a combination of one CpG in the gene promoter (CpG07504598) and one CpG in the gene body (CpG12922093) of *DFNA5*, which was able to discriminate between breast cancer and normal breast samples (AUC = 0.93). This model was externally validated in three independent datasets. Moreover, we showed that estrogen receptor state is associated with *DFNA5* methylation and expression. Finally, we were able to find a significant effect of *DFNA5* gene body methylation on a 5-year overall survival time.

**Conclusions:**

We conclude that *DFNA5* methylation shows strong potential as detection and prognostic biomarker for breast cancer.

**Electronic supplementary material:**

The online version of this article (10.1186/s13148-018-0479-y) contains supplementary material, which is available to authorized users.

## Background

Breast cancer is the most frequent cancer among women, with nearly 1.67 million new cases diagnosed in 2012 [1]. It is a heterogeneous disease consisting of two main histological subtypes, ductal and lobular adenocarcinomas, that differ with respect to clinical presentation, morphological and molecular features, and clinical behavior [2–5]. Breast cancer ranks as the most frequent and second most frequent cause of cancer-related mortality in women in less developed and more developed regions, respectively [1]. The high mortality can partly be explained by late detection. Therefore, the World Health Organization emphasizes that: “early diagnosis in order to improve breast cancer outcome and survival remains the cornerstone of breast cancer control” [6]. Until now, the only early detection method for breast cancer with proven efficacy is mammography screening. Although there is evidence that mammography screening programs can reduce breast cancer mortality, there is a narrow balance of benefits compared with harms, particularly in respect to overdiagnosis and overtreatment [7]. Therefore, identification of new highly specific biomarkers enabling early detection is much needed.

Over the last years, increasing evidence for a role of epigenetic mechanisms in (breast) cancer development and progression has been obtained. Inactivation of tumor suppressor genes through DNA methylation and histone modifications, together with global hypomethylation leading to increased genomic instability, are hallmarks of cancer [8–14]. Moreover, epigenetic modifications are believed to be early events in breast cancer development due to their presence even in carcinoma in situ lesions, which makes them very suitable as early detection biomarkers [15–21]. The identification of methylation markers that are sensitive and specific for (breast) cancer may contribute to early detection. We hypothesize that *DFNA5* may be a valuable epigenetic biomarker, based upon large differences in *DFNA5* methylation between breast cancer and healthy breast tissues, strong indications for its role as tumor suppressor gene, and its function in regulated cell death.

The *deafness*, *autosomal dominant 5* (*DFNA5*; also known as *ICERE* or *GSDME*) gene was identified in our lab in 1998 [22]. We have demonstrated that DFNA5 has the capacity to induce regulated cell death [23–25]. Recently, DFNA5 has been in the spotlight as Rogers et al. showed that caspase-3 cleaves DFNA5 to generate a necrotic DFNA5-N fragment. This fragment targets the plasma membrane and permeabilizes it by forming DFNA5 pores. Thereby, DFNA5 induces secondary necrosis, which is a lytic and inflammatory phase that occurs when apoptotic cells are not scavenged [26]. Soon after Rogers’ publication, several other papers pointed towards an important role for DFNA5 in secondary necrosis and its possible pathophysiological and therapeutic implications, especially in cancer [27–30]. Moreover, genomic methylation screens unveiled *DFNA5* as a possible tumor suppressor gene [31–33]. Epigenetic silencing through *DFNA5* methylation was previously shown in gastric [31], colorectal [32, 34], and breast cancer [35] on a limited number of samples. Recently, we performed methylation analysis on four CpGs in the *DFNA5* promoter region using bisulphite pyrosequencing on 123 primary breast adenocarcinomas, 16 histologically normal breast tissues adjacent to the tumor, and 24 breast reduction tissues from women without cancer [36] (Fig. 2). Significantly higher methylation percentages were seen in the adenocarcinoma samples compared to those in the healthy breast reduction samples. A receiver operating characteristic (ROC) curve for *DFNA5* methylation showed a sensitivity of 61.8% for the detection of breast cancer with a specificity of 100% [36]. We concluded that *DFNA5* methylation shows strong potential as biomarker for detection of breast cancer. However, the number of samples, the number of CpGs analyzed, the correlation with *DFNA5* expression, and the associations with survival parameters were still limited.

**Fig. 1:**
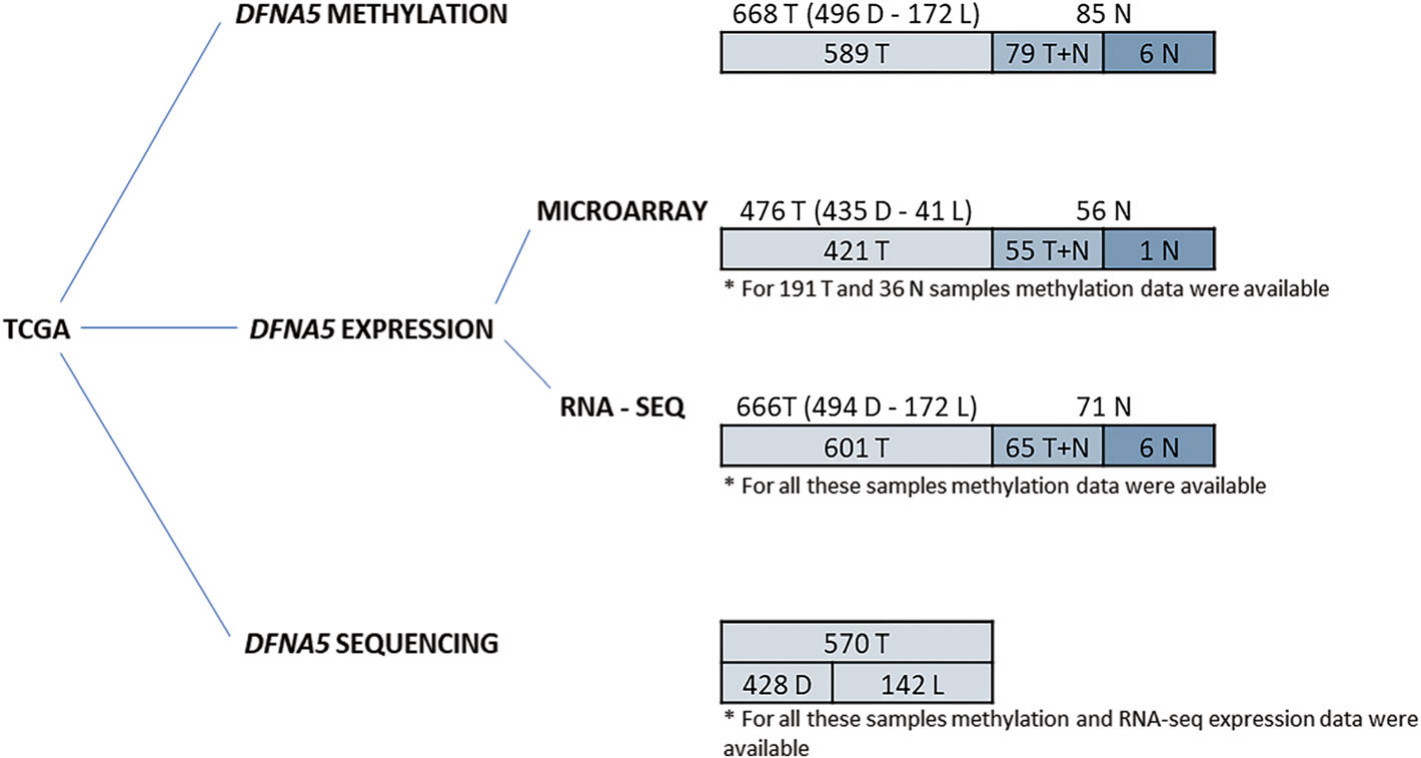
The number of samples for *DFNA5* methylation, expression, and sequencing. *DFNA5* methylation data were available for 668 unique, primary, untreated, female, well-characterized breast adenocarcinomas (T) (496 ductal (D)–172 lobular (L)) and 85 unique, untreated, female histologically normal breast tissues at a distance of the tumor (N). For 79 of these patients, both a tumor and a normal breast sample were available (paired samples (T+N)). *DFNA5* microarray expression data were available for 476 T (435 D–41 L) and 56 N. For 55 of these patients, both a tumor and a normal breast sample were available (T+N). For 191 of these T and 36 of these N, both *DFNA5* methylation and expression data were available. *DFNA5* RNA-seq expression data were available for 666 T (494 D–172 L) and 71 N. For 65 of these patients, both a tumor and a normal breast sample were available (T+N). For all these samples, also methylation data were available. *DFNA5* sequencing data were available for 570 T (428 D–142 L). For all these samples, methylation and RNA-seq expression data were also available

In this study, we aimed to analyze *DFNA5* methylation and expression in the largest breast adenocarcinoma patient cohort to date (Fig. 1) using publicly available data from The Cancer Genome Atlas (TCGA) in order to further unravel the role of *DFNA5* as detection and/or prognostic marker in breast cancer [37].

## Methods

### Study population and tissue samples

All analyses in this manuscript were performed using TCGA data. We selected female, ductal and lobular breast samples that were not neoadjuvantly treated for our analyses. *DFNA5* methylation, expression, and sequencing data were downloaded from the TCGA data portal using an in-house developed Python script. The number of samples in each group are shown in Fig. 1. Characteristics of the study populations are shown in Table 1. The mean age of the patients was 57.8 ± 13.0 years (range 26–90 years). A batch number is assigned to a set of related analytes from the same disease that has been distributed to one of the Genome Sequencing Centers.

**Table 1:** Clinicopathological features of the TCGA breast adenocarcinomas

Clinicopathological parameter	Methylation (*N* = 668)		Expression–microarray (*N* = 476)		Expression–RNA-seq (*N* = 666)	
	Number (%)	*P* value (range)	Number (%)	*P* value	Number (%)	*P* value
ER		2.2 × 10^−22^–0.049 (20/22 CpGs)		0.038		3.3 × 10^− 3^
ER+	490 (73.3)		362 (76.0)		488 (73.3)	
ER−	142 (21.3)		107 (22.5)		142 (21.3)	
Unknown	36 (5.4)		7 (1.5)		36 (5.4)	
PR		9.7 × 10^−15^–3.0 × 10^−3^ (15/22 CpGs)		N.S.		N.S.
PR+	428 (64.1)		310 (65.1)		427 (64.1)	
PR−	201 (30.1)		158 (33.2)		200 (30.0)	
Unknown	39 (5.8)		8 (1.7)		39 (5.9)	
*HER2*		0.023 (1/22 CpGs)		N.S.		N.S.
*HER2*+	32 (4.8)		45 (9.4)		32 (4.8)	
*HER2*−	195 (29.2)		166 (34.9)		195 (29.3)	
Unknown	441 (66.0)		265 (55.7)		439 (65.9)	
Tumor stage		1.9 × 10^−4^–0.020 (5/22 CpGs)		N.S.		N.S.
I	109 (16.3)		83 (17.5)		107 (16.1)	
II	378 (56.6)		269 (56.5)		378 (56.8)	
III	166 (24.9)		99 (20.8)		166 (24.9)	
IV	9 (1.3)		12 (2.5)		9 (1.3)	
Unknown	6 (0.9)		13 (2.7)		6 (0.9)	
Histological diagnosis		1.0 × 10^−4^–0.038 (10/22 CpGs)		4.2 × 10^−4^		3.2 × 10^−4^
Ductal	496 (74.3)		435 (91.4)		494 (74.2)	
Lobular	172 (25.7)		41 (8.6)		172 (25.8)	

Important clinicopathological parameters, such as ER status, PR status, *HER2* status, tumor stage (I–IV), and histological diagnosis are reported for the breast adenocarcinomas. The numbers of adenocarcinomas in each category are reported for the methylation and expression (both microarray and RNA-seq) dataset. The significant *p* values for the association analysis with either *DFNA5* methylation, *DFNA5* microarray expression, or *DFNA5* RNA-seq expression are reported

N.S. not significant

#### Methylation data

**Fig. 2:**
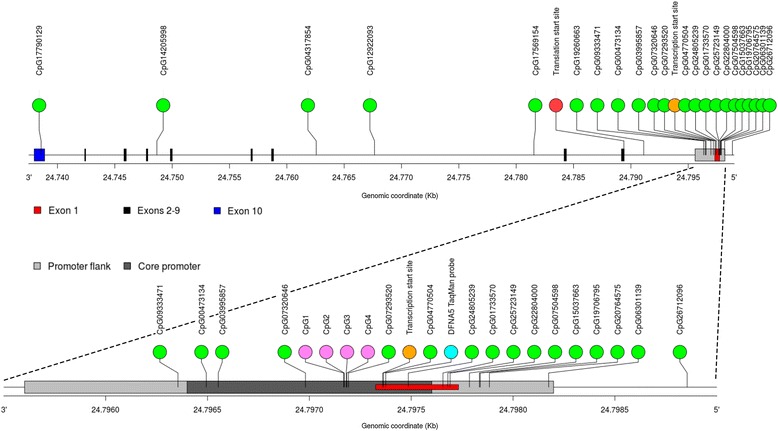
The *DFNA5* gene with annotation of the 22 CpGs. The 10 exons and the promoter and gene body region of the *DFNA5* gene are indicated. These annotations (GRCh37) were made based on the “Regulatory build of the *DFNA5* gene” in Ensembl. We considered the core promoter (7:24796400-24797601) together with the flanking regions (7:24795602-24798199) as the putative promoter of *DFNA5*. On basis of this annotation, six CpGs are located in the *DFNA5* gene body, 15 CpGs are located in the *DFNA5* promoter, and one CpG is located upstream of the *DFNA5* promoter. Using these annotations, CpG06301139 strictly belongs to the promoter of *DFNA5*. However, in this study, we considered CpG06301139 still part of the upstream promoter region because the methylation pattern is clearly different from the other promoter CpGs and it is located 24 base pairs from the border of the flanking region of the *DFNA5* promoter. In addition to the 22 CpGs analyzed in this study (green dots), the four CpGs analyzed in our previous study (pink dots, [36]) and the TaqMan probe (6FAM 5′-ATTCGACCCCGCGAAAAAACGCCGCT-3′-TAMRA) of the study of Kim et al. (blue dot, [35]) are annotated. The transcription start site and the translation start site are indicated with an orange dot and a red dot, respectively

TCGA methylation data (level 3) were obtained using Infinium HumanMethylation450 BeadChip® microarrays (Illumina Inc., San Diego, CA, USA). Twenty-two different CpGs throughout the *DFNA5* gene were available. The genomic coordinates of the CpGs are based on GRCh37 (Fig. 2). All methylation values are expressed as *β* values, which is the ratio of the methylated probe intensity to the overall intensity (the sum of methylated and unmethylated probe intensities).

#### Expression data

TCGA expression data (level 3) were obtained using both Agilent 244K Custom Gene Expression G4502A-07® microarrays (Agilent, Santa Clara, CA, USA) and the IlluminaHiSeq_RNASeqV2 platform (Illumina, San Diego, CA, USA). The Agilent microarray contains two probes for *DFNA5* (A_23_P82448 [36.3:chr.7:24705001-24705060] and A_23_P82449 [36.3:chr.7:24705092-24705151]), covering the three most abundant *DFNA5* transcripts (NM_004403.2, NM_001127454.1, and NM_001127453.1). All microarray expression values are expressed as log2 fc (fold change) relative to the Universal Human Reference RNA (Stratagene). The *DFNA5* transcript NM_004403.2 was most abundant in the ribonucleic acid sequencing (RNA-seq) data. The expression of the other transcripts was negligible. RNA-Seq by Expectation Maximization (RSEM) was used as the algorithm for quantifying transcript abundances from RNA-seq data [38]. All RNA-seq expression values are log2 transformed.

#### Clinicopathological parameters

We selected the following clinicopathological parameters from the TCGA Clinical Patient Data files to perform association analyses: age at diagnosis, estrogen receptor (ER) status determined by immunohistochemistry (IHC) (positive–negative), progesterone receptor (PR) status determined by IHC (positive–negative), *human epidermal growth factor receptor 2* (*HER2*) status determined by fluorescent in situ hybridization (FISH) (positive – negative), American Joint Committee on Cancer (AJCC) pathological tumor stage (I–IV), and histological diagnosis (ductal–lobular) (Table 1).

### Validation datasets

Three additional methylation datasets were downloaded from the Gene Expression Omnibus (GEO) [39] (GEO accession numbers: GSE52865, GSE69914, and GSE60185). The number of samples used from each dataset are shown in Additional file 1: Table S14.

### Statistical analysis

All statistical analyses were carried out using the statistical package R, version 3.1.2 [40]. All *p* values are two-sided, and *p* values ≤0.05 were considered statistically significant.

To account for possible batch effects, association tests accounted for the non-independence between individuals from the same batch by fitting a linear mixed model including a random effect for batch number. The significance of the fixed effects was tested via the F-test with a Kenwardroger correction for the number of degrees of freedom. Throughout the regression models, age was accounted for as a covariate, but it was removed from the model if the effect on the outcome was not significant.

Linear mixed models were fit using the lme4 package [41]. Cox proportional hazard models were fit using the survival package [42], to model 5-year overall survival (OS) time based upon either *DFNA5* methylation or *DFNA5* expression (microarray or RNA-seq), accounting for age. Models with separate baseline hazards for the four tumor stages were fit. Individuals who died without a tumor were considered “lost to follow-up”. Moreover, individuals who died 5 years (1826 days) or more after first diagnosis were censored. For these individuals, follow-up time was set to 1826 days. False discovery rates (FDRs) were calculated using the q-value package [43]. In the quantile-quantile (Q-Q) plots, the distribution of the 22 observed *p* values is compared to the uniform distribution (U(0,1)), which is expected in the absence of any true association signal. The relative contribution of the methylation of a CpG to 5-year OS time was estimated by comparing the concordance between two Cox proportional hazard models: one baseline model with only tumor stage and age as covariates, and five models to which one of the five CpGs were added as explanatory variable.

## Results

### *DFNA5* methylation and expression in primary breast adenocarcinomas and paired histologically normal breast tissues at a distance of the tumor

**Fig. 3:**
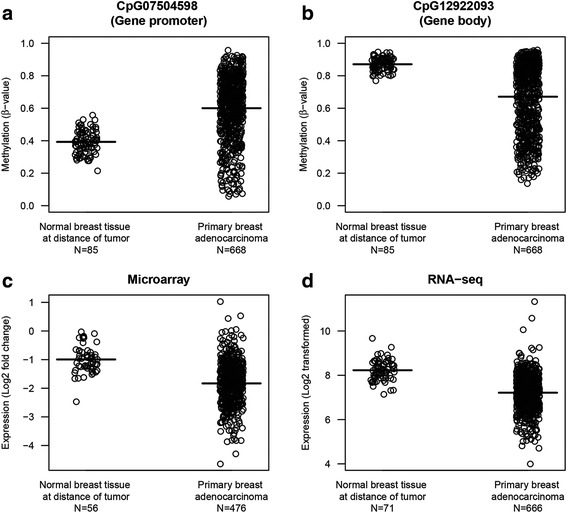
*DFNA5* methylation and expression in breast adenocarcinomas and normal breast tissues. Panels **a** and **b** *DFNA5* methylation values are reported for the primary breast adenocarcinomas and normal breast tissues at a distance of the tumor in two CpGs, as a typical example of *DFNA5* methylation. Panels **c** and **d** *DFNA5* expression values are reported for microarray (panel **c**) and RNA-seq (panel **d**) experiments for both the primary breast adenocarcinomas and normal breast tissues at a distance of the tumor. Negative expression values for the microarray data indicate a downregulation relative to the Universal Human Reference RNA (Stratagene). The means are indicated with a bold line

**Fig. 4:**
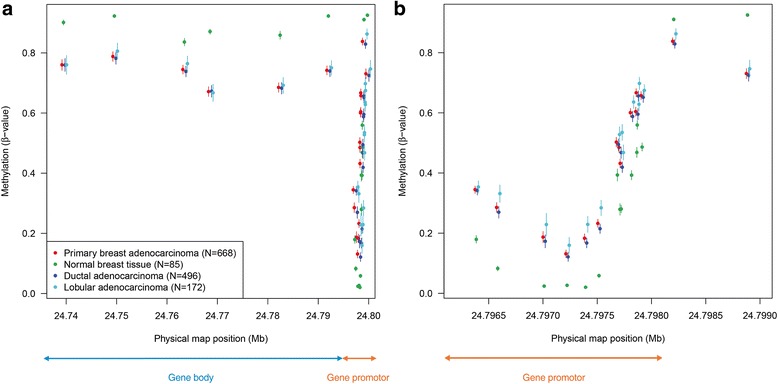
Physical map of the 22 CpGs in *DFNA5*, plotting chromosomal location versus average methylation values. Different subgroups (tumor vs normal; ductal vs lobular) have been plotted. Both the gene body and the putative gene promoter region of *DFNA5* are indicated. These figures clearly indicate that normal samples have higher methylation values in the gene body, compared to tumor samples (panel **a**). The opposite is true for the promoter of *DFNA5* (panel **b**). Moreover, the lobular breast adenocarcinomas showed higher mean *DFNA5* methylation values compared to the ductal breast adenocarcinomas in or upstream from the putative gene promoter region. Panel **b** (gene promoter region) is a magnification of panel **a**

*DFNA5* methylation values were plotted for the primary breast adenocarcinomas and normal breast tissues in two CpGs, one in the gene promoter (CpG07504598) and one in the gene body (CpG12922093), as typical example of *DFNA5* methylation (Fig. 3a, b). The mean *DFNA5* methylation for CpG07504598 was 0.60 (95% CI 0.58–0.62) for the breast adenocarcinomas and 0.39 (95% CI 0.38–0.40) for the normal breast tissues (Fig. 3a). For *DFNA5* CpG12922093, the mean methylation was 0.67 (95% CI 0.65–0.69) for the breast adenocarcinomas and 0.87 (95% CI 0.86–0.88) for the normal breast tissues (Fig. 3b). Using a paired samples *t* test, *DFNA5* methylation was investigated in 79 paired breast adenocarcinoma and normal breast samples (Additional file 1: Figure S1A, B). Our analysis showed a significant difference between primary tumor and paired normal breast samples for all 22 CpGs (Additional file 1: Table S1). Overall, breast adenocarcinomas showed higher methylation of CpGs located in the gene promoter compared to normal breast samples. The opposite is true for CpGs located in the gene body (Fig. 4).

Moreover, *DFNA5* expression was significantly lower in breast adenocarcinomas compared to normal breast samples*.* The mean *DFNA5* microarray expression (log2 fold change (fc)) was − 1.8 (95% CI − 1.9 to − 1.8) for the breast adenocarcinomas and − 0.99 (95% CI − 1.1 to − 0.87) for the normal breast tissues (Fig. 3c). Microarray data showed an observed mean log2 fc difference in *DFNA5* expression between normal and tumor sample within the same patient of 0.75 (95% CI 0.53–0.96) (*p* = 1.8 × 10^−09^) (Additional file 1: Figure S1C). The mean *DFNA5* RNA-seq expression (log2) for the breast adenocarcinomas was 7.2 (95% CI 7.2–7.3) and for the normal breast tissues the mean *DFNA5* RNA-seq expression was 8.2 (95% CI 8.1–8.3) (Fig. 3d). The observed mean log2 difference in *DFNA5* RNA-seq expression between normal and tumor sample within the same patient was 0.90 (95% CI 0.69–1.12) (*p* = 2.2 × 10^−16^) (Additional file 1: Figure S1D).

We also investigated the correlation between *DFNA5* microarray and RNA-seq expression data for both 189 breast adenocarcinomas and 35 normal breast samples, for which both microarray and RNA-seq *DFNA5* expression data were available. The results are shown in Additional file 1: Figure S2.

### Physical mapping of the 22 CpGs in the *DFNA5* gene

We plotted the average *DFNA5* methylation for all 22 CpGs against their physical map position on chromosome 7 for both primary breast adenocarcinomas and histologically normal breast tissues at a distance of the tumor, and ductal and lobular adenocarcinomas (Fig. 4). A clustering of the methylation values at the different positions could be observed. On the basis of these *DFNA5* methylation values, a clear difference exists between the gene body and gene promoter region. The first six CpGs are located in the gene body region, where the mean *DFNA5* methylation values of the cancer samples were lower than those of the normal samples. On the other hand, the 14 CpGs which are located in the putative gene promoter region had a higher methylation value in the cancer compared to that in the normal samples. For the last two CpGs this pattern reversed again. We believe that these CpGs are located upstream of the putative gene promoter region (Fig. 2).

### Association between *DFNA5* methylation and expression

We examined whether *DFNA5* methylation is associated with *DFNA5* expression, first by calculating the spearman correlation coefficient for *DFNA5* expression and methylation for each of the individual 22 CpGs and secondly by fitting a stepwise backward linear regression of the expression data on all 22 CpG methylation values for both breast adenocarcinoma and normal breast samples. All analyses were performed with the microarray and RNA-seq expression data.

First, Spearman correlation coefficients were calculated for samples of which both *DFNA5* methylation and expression data were available (Fig. 1). None of the correlations were strong (all < 0.35), which implies that the methylation status of none of the CpGs alone allows an accurate prediction of the *DFNA5* expression, neither microarray nor RNA-seq (data not shown).

To predict the expression based upon the methylation of one or more CpGs, multiple linear regression models were fit. For the breast adenocarcinomas, about 20% of the variance in *DFNA5* expression is attributable to *DFNA5* methylation (microarray: Additional file 1: Table S2; RNA-seq: Additional file 1: Table S3). For the normal breast samples, a regression model was fit for the microarray expression data only (Additional file 1: Table S2). For the RNA-seq expression data, none of the 22 CpGs showed a significant association with *DFNA5* expression in the normal samples, and therefore no multiple regression model could be built (data not shown). For the normal samples, these results are somewhat divergent and therefore it is hard to estimate the contribution of *DFNA5* methylation on the expression level of these samples. In general, we conclude there is no clear association between *DFNA5* methylation and expression.

### *DFNA5* methylation and expression as detection biomarker for breast cancer

We investigated whether a specific combination of the 22 CpGs analyzed can be used as detection biomarker for breast cancer. Therefore, we analyzed which CpGs discriminate best between primary breast adenocarcinomas (*N* = 668) and normal breast samples (*N* = 85). Using stepwise logistic regression, we searched for a model to predict the tumor status of a given tissue using the area under the curve (AUC) as a criterion. Several models reached an AUC in the range of 0.93–0.95. Among these models, we chose a model with high specificity. The model including one CpG in the gene body (CpG12922093) and one CpG in the gene promoter (CpG07504598) as predictors had a tenfold cross-validated AUC of 0.93 (95% CI 0.92–0.95). With the methylation (*β*) values of these two CpGs, the predicted probability can be calculated:

$$ \mathrm{predicted}\ \mathrm{probability}={e}^{\left(7.49-10.77\ast \mathrm{CpG}12922093+6.33\ast \mathrm{CpG}07504598\right)}/1+{e}^{\left(7.49-10.77\ast \mathrm{CpG}12922093+6.33\ast \mathrm{CpG}07504598\right)} $$

**Fig. 5:**
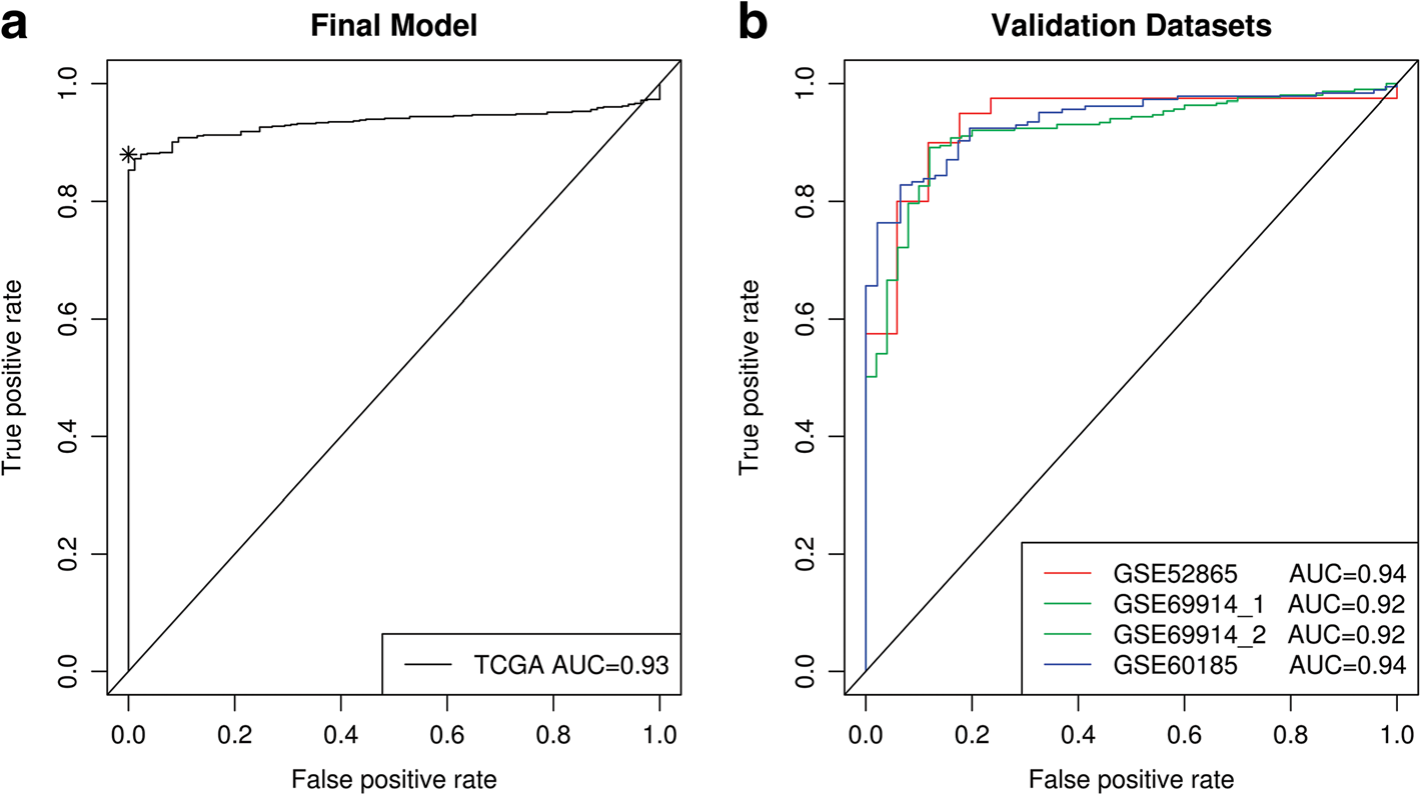
*DFNA5* methylation as biomarker for breast cancer. Panel **a** One CpG in the gene body (CpG12922093) and one CpG in the gene promoter (CpG07504598) were used as predictors. Sensitivity and specificity at various cutoff values for our dataset are shown. The diagonal line represents the line of no discrimination between breast adenocarcinoma and normal breast samples. The predicted probability (cutoff) of 0.87 is indicated with an asterisk. Panel **b** Three independent datasets, originating from GEO (GSE52865, GSE69914, and GSE60185), were used to validate our model. Two analyses were performed using GSE69914. First, the analysis was performed on 305 breast cancers and 50 normal breast tissues from healthy women (GSE69914_1). Secondly, 305 breast cancers and 42 normal breast tissues, adjacent to the tumor were used to perform the analysis (GSE69914_2). The AUCs for both are almost identical, and the curves are fully overlapping

Sensitivities and specificities at the different cutoff values for the predicted probabilities are shown in Fig. 5. At a predicted probability of 0.87, a sensitivity of 85.3% for detection of breast adenocarcinomas is reached without false positives, with an overall accuracy of 87.0% in our dataset. To further externally validate our findings, we applied our model to three independent methylation datasets to predict the tumor status of a given tissue (Additional file 1: Table S14). We were able to successfully predict the tumor status of the tissues in all three datasets with AUCs comparable to that of the original TCGA dataset (Fig. 5). In general, the model exhibited a high predictive power and good generalizability over different datasets.

Moreover, we investigated whether *DFNA5* expression (either microarray or RNA-seq) could be a detection biomarker for breast cancer. For *DFNA5* microarray expression, we obtained a ROC with a tenfold cross-validated AUC of 0.82 (95% CI 0.78–0.87) (Additional file 1: Figure S3A). For the *DFNA5* RNA-seq expression, a ROC with a tenfold cross-validated AUC of 0.88 (95% CI 0.85–0.91) was reached (Additional file 1: Figure S3B).

### *DFNA5* methylation and expression in ductal breast adenocarcinomas compared to lobular breast adenocarcinomas

We investigated the difference between ductal and lobular breast adenocarcinomas for both *DFNA5* methylation and expression (either microarray or RNA-seq), by fitting a linear mixed model. In 10 out of 22 CpGs, the lobular adenocarcinomas showed significantly higher mean *DFNA5* methylation values compared to the ductal adenocarcinomas (Table 1; Fig. 4; Additional file 1: Table S4). All of these 10 CpGs are located in (9/10) or upstream (1/10) from the putative gene promoter region.

Moreover, the lobular adenocarcinomas had a significantly higher *DFNA5* expression compared to the ductal adenocarcinomas (Table 1). For the microarray expression values, the mean log2 fc *DFNA5* expression for the ductal adenocarcinomas was − 1.86 (95% CI − 1.87 to − 1.86) and for the lobular adenocarcinomas − 1.48 (95% CI − 1.52 to − 1.45). For the RNA-seq expression values, the mean log2 *DFNA5* expression for the ductal adenocarcinomas was 7.15 (95% CI 7.08–7.23) and for the lobular adenocarcinomas 7.39 (95% CI 7.29–7.50).

### Associations between *DFNA5* methylation or expression and clinicopathological parameters

We tested the effect of four clinicopathological parameters (ER status, PR status, *HER2* status, or tumor stage (I–IV)) on *DFNA5* methylation or expression, both on microarray and RNA-seq data, by fitting a linear mixed model (Table 1). Association analysis showed a significant association between ER status and *DFNA5* methylation in 20/22 CpGs (Additional file 1: Table S5) and *DFNA5* expression, both with the microarray and the RNA-seq data. The *DFNA5* expression was higher in the ER− compared to the ER+ breast adenocarcinomas (Additional file 1: Table S6). In 15/22 CpGs, a significant association between PR status and *DFNA5* methylation was observed (Additional file 1: Table S5). Only methylation of CpG04317854 was significantly associated with *HER2* amplification (Additional file 1: Table S5). Furthermore, tumor stage was significantly associated with *DFNA5* methylation in 5 out of 22 CpGs (Additional file 1: Table S7). There were only nine patients with a stage IV breast adenocarcinoma; these were not included in the analysis. None of these clinicopathological parameters (PR, *HER2*, and tumor stage) showed a significant association with *DFNA5* expression, with neither microarray nor with RNA-seq data.

### Associations between *DFNA5* methylation or expression and 5-year overall survival

Overall survival (OS) was investigated by fitting Cox proportional hazard models over a 5-year period to determine the prognostic value of *DFNA5* methylation or expression, using either microarray or RNA-seq data, in breast adenocarcinoma patients. Follow-up data were not available for all patients (Additional file 1: Table S8). Cox proportional hazard models were fit to model the survival time based upon either *DFNA5* methylation or *DFNA5* expression (microarray or RNA-seq). Models were fit on all breast adenocarcinoma patients, only the ductal, or only the lobular adenocarcinoma patients.

**Fig. 6:**
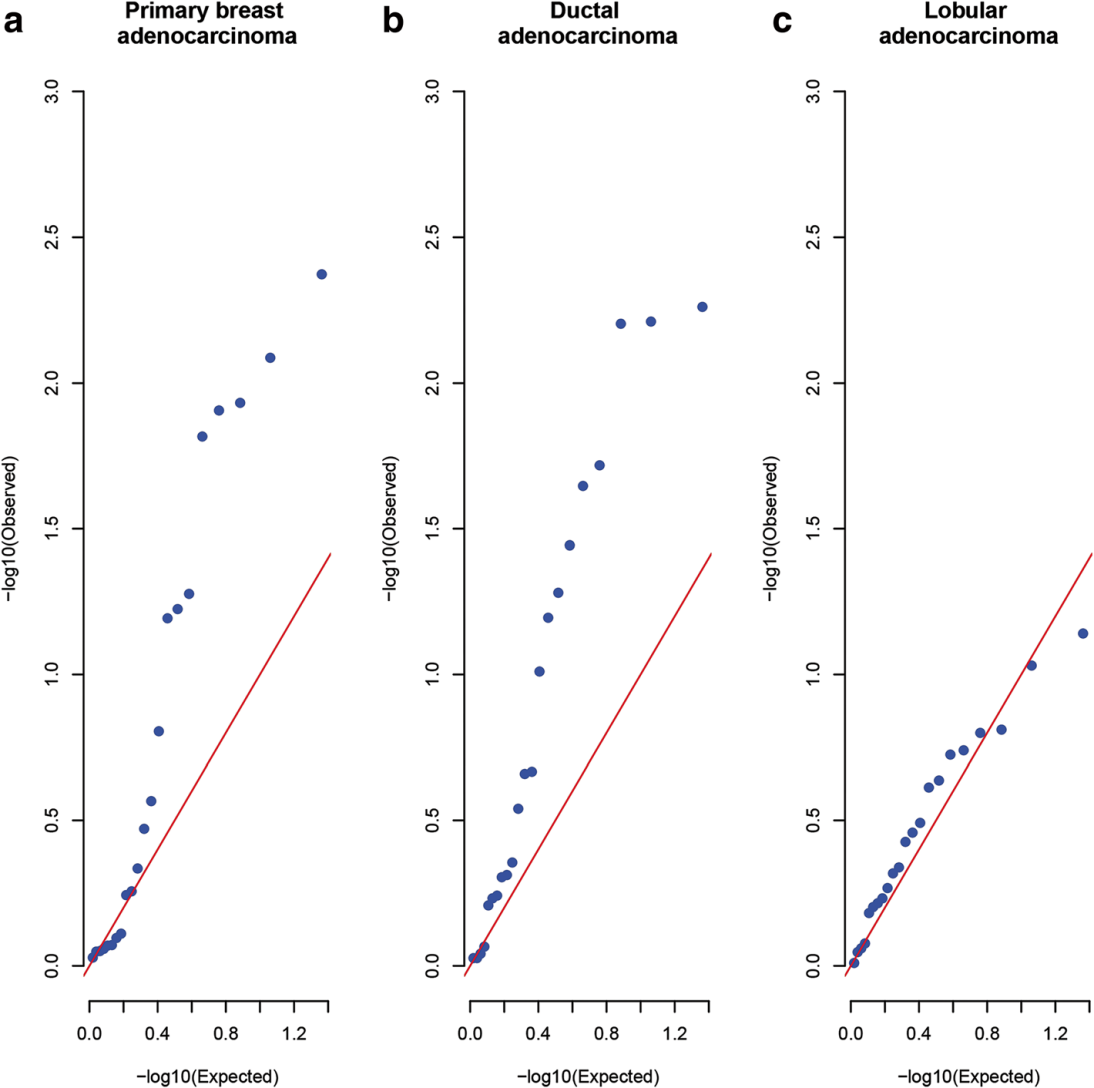
Q-Q plots of the 22 *p* values for the 5-year OS analysis. Under the null hypothesis of no association, *p* values follow a uniform distribution between 0 and 1. The diagonal line shows this expected distribution of *p* values. The points on the plot show the *p* values observed in the survival analysis. For all breast adenocarcinomas together (panel **a**) and only the ductal adenocarcinomas (panel **b**), the presence of many points above the diagonal line indicates a substantial enrichment in low *p* values. The *p* values in the lobular subset (panel **c**) are closely following the expected distribution of the *p* values, indicating no enrichment in low *p* values there

Survival analysis on all breast adenocarcinoma patients showed a significant association between 5-year OS time and *DFNA5* methylation in 5/22 CpGs (Table 2). Since a Bonferroni correction for multiple testing would not be appropriate due to the strong correlation in methylation between the CpG islands (data not shown), we tested for an enrichment in low *p* values using Q-Q plots (Fig. 6) and performed a false discovery rate (FDR) analysis (Additional file 1: Table S9). The Q-Q plot clearly indicates an increase in significant *p* values compared to the expected null distribution. Therefore, the FDR analysis shows that it is very likely that some of the significant *p* values represent genuine association signals. This suggests that the methylation of the CpGs as a whole contains information on 5-year OS time and strengthens the potential of *DFNA5* methylation as a prognostic marker. A very similar observation was made when studying the ductal adenocarcinoma patients only, with one additional significant CpG, located upstream from the putative gene promoter of *DFNA5* (Table 2). In the lobular adenocarcinoma patients, the enrichment of low *p* values was not observed, but it cannot be excluded that this is due to the lower number of observations in this latter subset (Table 2; Fig. 6; Additional file 1: Table S8).

**Table 2:** The effect of methylation of every of the 22 CpGs on 5-year OS time

CpG	Genomic coordinate (GRCh37)	All breast adenocarcinomas			Ductal adenocarcinomas			Lobular adenocarcinomas		
		Regression coefficient	S.E.	*P* value	Regression coefficient	S.E.	*P* value	Regression coefficient	S.E.	*P* value
CpG17790129	24738572	5.1	1.9	8.2 × 10^−3^ *	6.0	2.5	0.019 *	2.3	3.8	0.54
CpG14205998	24748668	5.1	2.1	0.015 *	4.9	2.1	0.023 *	13.9	11.9	0.24
CpG04317854	24762562	1.8	1.3	0.16	2.6	1.4	0.064	− 2.6	3.5	0.46
CpG12922093	24767644	3.1	1.2	0.012 *	3.8	1.4	6.2 × 10^−3^ *	− 1.9	4.2	0.66
CpG17569154	24781545	3.0	1.2	0.012 *	3.6	1.3	6.3 × 10^−3^ *	− 2.3	4.4	0.61
CpG19260663	24791121	5.3	1.9	4.2 × 10^−3^ *	5.7	2.0	5.5 × 10^−3^ *	7.5	6.3	0.23
CpG09333471	24796355	2.5	1.3	0.059	1.7	1.4	0.22	11.4	6.8	0.093
CpG00473134	24796494	0.81	1.4	0.57	0.78	1.6	0.62	4.3	4.8	0.37
CpG03995857	24796553	− 0.19	1.0	0.85	− 0.75	1.1	0.49	7.4	5.2	0.16
CpG07320646	24796981	− 0.49	0.84	0.55	− 1.3	1.0	0.22	7.0	5.3	0.19
CpG07293520	24797192	− 0.17	1.2	0.89	− 1.7	1.6	0.29	9.5	5.3	0.072
CpG04770504	24797363	− 1.3	1.2	0.27	− 2.4	1.4	0.098	11.7	8.8	0.18
CpG24805239	24797486	− 0.19	1.2	0.87	− 0.91	1.3	0.49	6.4	4.5	0.16
CpG01733570	24797656	0.97	1.0	0.34	0.77	1.0	0.44	3.9	3.9	0.32
CpG25723149	24797680	0.14	1.0	0.89	0.18	1.0	0.86	2.2	4.1	0.59
CpG22804000	24797691	− 0.092	1.1	0.93	0.087	1.1	0.94	0.51	3.9	0.90
CpG07504598	24797786	− 0.31	1.1	0.77	− 0.13	1.1	0.91	0.87	4.2	0.83
CpG15037663	24797835	− 0.21	1.1	0.85	− 0.086	1.1	0.94	3.4	4.8	0.48
CpG19706795	24797839	0.29	1.2	0.80	0.67	1.2	0.58	0.13	4.3	0.98
CpG20764575	24797884	− 0.77	1.1	0.46	− 0.60	1.1	0.57	0.84	5.0	0.87
CpG06301139	24798175	4.2	2.3	0.064	5.0	2.6	0.052	2.9	5.9	0.63
CpG26712096	24798855	2.4	1.2	0.053	2.7	1.3	0.036 *	3.9	4.2	0.35

For each of the 22 CpGs, the effect size (= regression coefficient) with standard error (S.E.) and the *p* value (likelihood ratio test) are reported for the effect on 5-year OS time in all breast adenocarcinoma patients, only the ductal, or only the lobular carcinoma patients. CpG17790129–CpG19260663 are located in the gene body, CpG09333471–CpG20764575 are located in the putative gene promoter, and the last two CpGs, CpG06301139 and CpG26712096, are located upstream from the putative gene promoter

*Significant *p* values

Remarkably, the five CpGs with methylation values significantly associated with 5-year OS time are all located in the gene body region of *DFNA5*. Moreover, the positive regression coefficients indicate that higher methylation values are associated with a decrease in survival time (Table 2). The contribution of each of the five significant CpGs to 5-year OS time was investigated in a Cox proportional hazard frame work. Due to the limited number of patients in stages I and IV, this contribution could only be studied for stages II and III. For stage II, adding *DFNA5* methylation to the survival model lead to an increase in concordance of 7.0–11.1%, while for stage III, this increase in concordance was 4.9–11.0%, depending on which of the five CpGs was used (Additional file 1: Table S10). We conclude that the increase in concordance of the five significant CpGs to 5-year OS time was very similar. This is not surprising, since the methylation of the five significant CpGs (all located within the gene body) are strongly correlated (data not shown). Similar results are obtained for the ductal adenocarcinoma patients only (Additional file 1: Table S11).

Survival analysis showed no significant association between *DFNA5* expression and 5-year OS time, neither microarray nor RNA-seq, for all breast adenocarcinoma patients or ductal and lobular adenocarcinoma patients only (Additional file 1: Table S8).

## Discussion

In this study, we evaluated the potential use of *DFNA5* methylation and expression as detection and prognostic biomarker in breast cancer, on basis of data obtained from TCGA. *DFNA5* methylation was significantly different between primary breast adenocarcinomas and normal breast samples for all 22 CpGs analyzed. Overall, breast adenocarcinomas showed a higher *DFNA5* methylation in the putative gene promoter compared to normal breast samples, whereas in the gene body and upstream of the putative gene promoter, the opposite is true. We can conclude that *DFNA5* follows the classical cancer methylation paradigm of hypermethylation of the CpG island promoter and global genomic hypomethylation [8]. These results are in line with those obtained in our previous study [36] and the study of Kim et al. [35], where only *DFNA5* promoter methylation was analyzed and different CpGs were investigated using pyrosequencing and TaqMan-methylation-specific PCR (TaqMan-MSP), respectively (Additional file 1: Table S12). *DFNA5* expression was significantly lower in breast adenocarcinomas compared to normal breast samples, for both microarray and RNA-seq data. These results were in line with those obtained by Kim et al. [35] and Stoll et al. [27].

Despite the clear difference between primary breast adenocarcinomas and normal breast tissues for both *DFNA5* methylation and expression, no clear association between *DFNA5* methylation and expression could be found. In literature, it has already been demonstrated that the relationship between epigenetics and gene expression can be more ambiguous than previously thought [44]. Moreover, Stoll et al. also concluded that DNA hypermethylation did not affect the expression of *DFNA5* [27]. This is in contrast to the study of Akino et al. in gastric cancer [31]. However, Akino et al. analyzed the methylation of different CpGs in *DFNA5*, which are not present on the Infinium HumanMethylation450 BeadChip® microarrays that TCGA used. Perhaps it is possible that methylation of specific CpGs in *DFNA5* may be necessary to influence its expression. However, different reasons exist why no association could be found. One reason could be that current data do not allow to discriminate between *DFNA5* DNA hydroxymethylation from methylation [45, 46]. Another confounding factor could be the expression of microRNAs (miRNAs) that regulate *DFNA5* expression. Mir_3p and mir26b_5p are two miRNAs that may interfere with *DFNA5* expression [47, 48]. Expression data of both miRNAs were available in TCGA. However, no association between *DFNA5* expression and mir_3p or mir26b_5p expression could be found (data not shown). Another possibility could be the existence of deleterious somatic *DFNA5* variants occurring in the breast adenocarcinomas. Analysis of TCGA whole exome sequencing data revealed only five (of a total of 570) patients with a somatic *DFNA5* variation (3 missense and 2 silent variants) (Additional file 1: Table S13). This is in line with the observation that mutations in pro-necrotic genes, including *DFNA5*, are infrequent and that reduction in copy numbers are observed in less than 2% of breast cancers [27]. Moreover, other (epigenetic) factors, such as histone modifications, could possibly also have an impact on (*DFNA5*) gene expression. Another possibility is chemical modification of the RNA, which can also regulate the expression of genes, the so called epitranscriptome [49–51]. It is clear that gene expression is a complex process and the interplay between many different genetic, epigenetic, and epitranscriptomic factors determines the expression level of a gene [11, 52–55]. Lastly, tumor heterogeneity may also be a reason why no association between *DFNA5* methylation and expression could be found. The tissue slices used for methylation and expression analysis are not identical, as they originate from a different part of the tumor. Moreover, as the percentage of the tumor cells is never 100% (TCGA uses samples with at least 60% tumor cells), the ratio of tumor versus normal cells can differ between those slices.

A major result of this study is the identification of a combination of two CpGs, one CpG in the promoter (CpG07504598) and one CpG in the gene body (CpG12922093) of *DFNA5*, which was able to discriminate between primary breast adenocarcinomas and normal breast samples. The model with those two CpGs as predictors had a tenfold cross-validated AUC of 0.93. Moreover, our model was externally validated in three independent datasets from the GEO database. The AUC values for these datasets were very similar to that of the original dataset, which confirms the validity of our model and its generalizability over external cohorts. All together, these results suggest a strong potential for *DFNA5* methylation as biomarker for the detection of breast cancer.

We found that *DFNA5* methylation was significantly higher in 10 out of 22 CpGs analyzed in lobular compared to ductal adenocarcinomas. Remarkably, those 10 CpGs are all located in or upstream of the putative gene promoter region and not in the gene body of *DFNA5*. Despite the higher *DFNA5* promoter methylation in the lobular adenocarcinomas, the *DFNA5* expression was also significantly higher in the lobular compared to the ductal adenocarcinomas.

We analyzed the association of *DFNA5* methylation and expression with four clinicopathological parameters. In line with the previous study of Thompson and Weigel [56], an inverse correlation between ER status and *DFNA5* expression could be found. Moreover, *DFNA5* methylation was also significantly associated with ER status in 20 out of 22 CpGs. *DFNA5* methylation in the putative gene promoter was always higher in the ER+ breast adenocarcinomas compared to the ER− breast adenocarcinomas and in the gene body region the opposite was true. This is in contrast to the study of Kim et al. [35] and our previous study [36] (Additional file 1: Table S12). However, in these studies, they analyzed a few CpGs which are not present on the Infinium HumanMethylation450 BeadChip® microarrays that TCGA used. Thompson and Weigel concluded that the pattern of *DFNA5* (*ICERE-1*) expression suggests that *DFNA5* may be involved in tumor biology specific to hormonally unresponsive breast cancers, and therefore, *DFNA5* expression may be a useful marker for this type of breast cancer [56].

Finally, despite the limited number of events, we were able to find a significant effect of methylation in the *DFNA5* gene body on 5-year OS time, for all breast adenocarcinoma patients together as well as for the ductal adenocarcinoma patients only (Additional file 1: Table S12). Remarkably, the five CpGs with a significant *p* value were all located in the gene body region of *DFNA5* and their positive regression coefficients indicate that higher methylation of these CpGs was associated with a decrease in survival time. The regulatory role of gene body methylation is still unclear, but could prevent spurious transcription initiation, may promote (alternative) splicing, or represent a higher order chromatin topologically associating domain to guide regulatory elements to the *DFNA5* promoter [52, 57–60]. Among those five CpGs located in the gene body, the most significant association with 5-year OS time was found for CpG19260663 in all breast adenocarcinoma patients together as well as in the ductal adenocarcinoma patients only. From the concordance tables, we can conclude that, in addition to the age of the patient, *DFNA5* gene body methylation has an added value of around 9% to predict 5-year OS time. The enrichment in low *p* values, shown in Q-Q plots and the FDR calculations, suggests that the methylation of the CpGs as a whole contain information on the survival time and strengthens the potential of *DFNA5* gene body methylation as a prognostic marker. Large prospective studies, with a homogeneous breast adenocarcinoma population (in terms of treatment), are needed to confirm the prognostic role of *DFNA5* gene body methylation in breast adenocarcinoma. The effect of *DFNA5* expression on 5-year OS time was not significant, corroborating previous findings [27].

## Conclusions

We conclude that *DFNA5* methylation shows strong potential as detection and prognostic biomarker for breast cancer. In order to evaluate the potential of *DFNA5* methylation as early biomarker, the analysis of in situ carcinoma samples could be a good strategy [15–21]. A next step to further investigate and develop *DFNA5* methylation as biomarker for breast cancer could be the analysis of *DFNA5* methylation in liquid biopsies. Several studies have provided proof of principle for the detection of promoter hypermethylation of tumor-derived DNA in liquid biopsies [61–66]. Using liquid biopsies, *DFNA5* methylation has the potential to be a suitable low invasive detection and prognostic biomarker for breast cancer.

## Additional files

Additional file 1: **Table S1.** Mean difference in *DFNA5* methylation between the paired tumor and normal breast sample in 79 patients for every of the 22 CpGs. **Figure S1.** *DFNA5* methylation (in the gene promoter and in the gene body) and expression (microarray and RNA-seq) in paired tumor and normal breast samples. **Figure S2.** Correlation between microarray and RNA-seq expression data. **Table S2.** Stepwise linear regression models of *DFNA5* microarray expression on *DFNA5* methylation for both breast adenocarcinoma and normal breast samples. **Table S3.** Stepwise linear regression model of *DFNA5* RNA-seq expression on *DFNA5* methylation for the breast adenocarcinomas. **Figure S3.** *DFNA5* expression as biomarker for breast adenocarcinomas. **Table S4.** Mean *DFNA5* methylation for the ductal and the lobular breast adenocarcinomas for every of the 22 CpGs. **Table S5.** Mean *DFNA5* methylation for ER status, PR status, and *HER2* status for every of the 22 CpGs. **Table S6.** Mean *DFNA5* expression for ER+ and ER− breast adenocarcinomas. **Table S7.** Mean *DFNA5* methylation for the four tumor stages for every of the 22 CpGs. **Table S8.**Vital status of the breast adenocarcinoma patients after 5 years of follow-up. **Table S9.** False discovery rate (FDR) for 5-year OS analysis on all breast adenocarcinomas and ductal breast adenocarcinomas. **Table S10.** Concordance for 5-year OS analysis on all breast adenocarcinomas. **Table S11.** Concordance for 5-year OS analysis on ductal breast adenocarcinomas. **Table S12.** Similarities and differences between three studies investigating *DFNA5* methylation in breast cancer. **Table S13.** Single nucleotide variants in the *DFNA5* gene with corresponding changes in the amino acid sequence of DFNA5. **Table S14.** Three methylation datasets from the Gene Expression Omnibus (GEO) for validation of our model to predict the tumor status. (DOCX 629 kb)

## Abbreviations

AJCC: American Joint Committee on Cancer
AUC: Area under the curve
*DFNA5*: *Deafness, autosomal dominant 5*
ER: Estrogen receptor
fc: Fold change
FDR: False discovery rate
FISH: Fluorescent in situ hybridization
GEO: Gene Expression Omnibus
*GSDME*: *Gasdermin E*
*HER2*: *Human epidermal growth factor receptor 2*
*ICERE*: *Inversely correlated with estrogen receptor expression*
IHC: Immunohistochemistry
miRNA: MicroRNA
N.S.: Not significant
OS: Overall survival
PR: Progesterone receptor
Q-Q: Quantile-quantile
RNA-seq: Ribonucleic acid sequencing
ROC: Receiver operating characteristic
RSEM: RNA-Seq by Expectation Maximization
S.E.: Standard error
TaqMan-MSP: TaqMan–methylation-specific polymerase chain reaction
TCGA: The Cancer Genome Atlas
