# Supplementary material for: Large-scale analysis of DFNA5 methylation reveals its potential as biomarker for breast cancer
Source: Clin Epigenetics. 2018 Apr 11;10:51. doi: 10.1186/s13148-018-0479-y (PMC5896072; doi:10.1186/s13148-018-0479-y)
Supplement: Supplementary file 1 — Table S1. Mean difference in DFNA5 methylation between the paired tumor and normal breast sample in 79 patients for every of the 22 CpGs. Figure S1. DFNA5 methylation (in the gene promoter and in the gene body) and expression (microarray and RNA-seq) in paired tumor and normal breast samples. Figure S2. Correlation between microarray and RNA-seq expression data. Table S2. Stepwise linear regression models of DFNA5 microarray expression on DFNA5 methylation for both breast adenocarcinoma and normal breast samples. Table S3. Stepwise linear regression model of DFNA5 RNA-seq expression on DFNA5 methylation for the breast adenocarcinomas. Figure S3. DFNA5 expression as biomarker for breast adenocarcinomas. Table S4. Mean DFNA5 methylation for the ductal and the lobular breast adenocarcinomas for every of the 22 CpGs. Table S5. Mean DFNA5 methylation for ER status, PR status, and HER2 status for every of the 22 CpGs. Table S6. Mean DFNA5 expression for ER+ and ER− breast adenocarcinomas. Table S7. Mean DFNA5 methylation for the four tumor stages for every of the 22 CpGs. Table S8.Vital status of the breast adenocarcinoma patients after 5 years of follow-up. Table S9. False discovery rate (FDR) for 5-year OS analysis on all breast adenocarcinomas and ductal breast adenocarcinomas. Table S10. Concordance for 5-year OS analysis on all breast adenocarcinomas. Table S11. Concordance for 5-year OS analysis on ductal breast adenocarcinomas. Table S12. Similarities and differences between three studies investigating DFNA5 methylation in breast cancer. Table S13. Single nucleotide variants in the DFNA5 gene with corresponding changes in the amino acid sequence of DFNA5. Table S14. Three methylation datasets from the Gene Expression Omnibus (GEO) for validation of our model to predict the tumor status. (DOCX 629 kb) [file 13148_2018_479_MOESM1_ESM.docx]

Additional file

Table S1. Mean difference in *DFNA5* methylation between the paired tumor and normal breast sample in 79 patients for every of the 22 CpGs.

| **CpG** | **Genomic coordinate (GRCh37)** | **Mean difference (T-N)** | **95% CI** | **P-value** |
| --- | --- | --- | --- | --- |
| CpG17790129* | 24738572 | -0.108 | -0.152 - -0.063 | 7.5*10^-6^ |
| CpG14205998* | 24748668 | -0.123 | -0.167 - -0.079 | 1.6*10^-7^ |
| CpG04317854* | 24762562 | -0.064 | -0.108 - -0.021 | 4.5*10^-3^ |
| CpG12922093* | 24767644 | -0.170 | -0.215 - -0.125 | 2.0*10^-11^ |
| CpG17569154* | 24781545 | -0.164 | -0.205 - -0.123 | 8.4*10^-12^ |
| CpG19260663* | 24791121 | -0.167 | -0.209 - -0.125 | 1.0*10^-12^ |
| CpG09333471* | 24796355 | 0.168 | 0.129 - 0.207 | 9.8*10^-14^ |
| CpG00473134* | 24796494 | 0.136 | 0.099 - 0.173 | 2.0*10^-11^ |
| CpG03995857* | 24796553 | 0.192 | 0.143 - 0.241 | 3.5*10^-12^ |
| CpG07320646* | 24796981 | 0.150 | 0.099 - 0.201 | 6.3*10^-8^ |
| CpG07293520* | 24797192 | 0.089 | 0.058 - 0.119 | 7.0*10^-8^ |
| CpG04770504* | 24797363 | 0.139 | 0.101 - 0.177 | 4.3*10^-11^ |
| CpG24805239* | 24797486 | 0.157 | 0.118 - 0.196 | 1.5*10^-12^ |
| CpG01733570* | 24797656 | 0.100 | 0.049 - 0.150 | 2.2*10^-4^ |
| CpG25723149* | 24797680 | 0.183 | 0.132 - 0.234 | 3.0*10^-10^ |
| CpG22804000* | 24797691 | 0.130 | 0.085 - 0.175 | 1.9*10^-7^ |
| CpG07504598* | 24797786 | 0.186 | 0.144 - 0.228 | 2.7*10^-13^ |
| CpG15037663* | 24797835 | 0.138 | 0.090 - 0.186 | 2.2*10^-7^ |
| CpG19706795* | 24797839 | 0.108 | 0.064 - 0.152 | 5.8*10^-6^ |
| CpG20764575* | 24797884 | 0.161 | 0.117 - 0.205 | 1.4*10^-10^ |
| CpG06301139* | 24798175 | -0.076 | -0.111 - -0.042 | 3.9*10^-5^ |
| CpG26712096* | 24798855 | -0.188 | -0.235 - -0.140 | 1.4*10^-12^ |

The mean differences in methylation between paired tumor and normal breast sample with 95% CI were reported for every of the 22 CpGs in the *DFNA5* gene. The CpGs with a significant p-value are marked with an asterisk. The CpGs in dark grey are located in the gene body, the CpGs in intermediate grey are located in the putative gene promoter and the last two CpGs, indicated in light grey, are located upstream from the putative gene promoter.


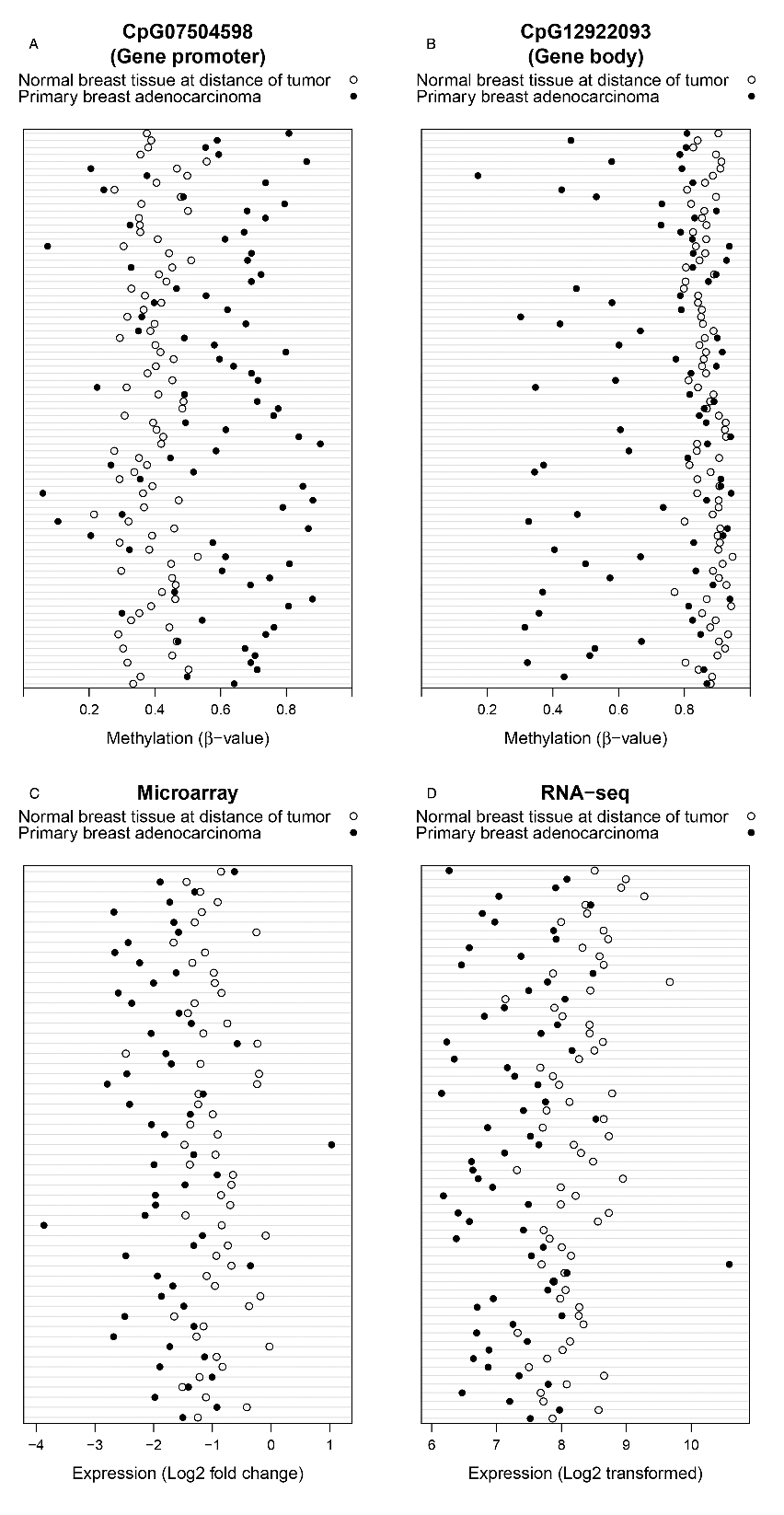


**Figure S1. *DFNA5* methylation (in the gene promoter and in the gene body) and expression (microarray and RNA-seq) in paired tumor and normal breast samples.** Panel A and B. *DFNA5* methylation values are reported for two CpGs, as a typical example of *DFNA5* methylation. Panel C and D. *DFNA5* expression values are reported for microarray (panel C) and RNA-seq (panel D). Negative expression values for the microarray data indicate a downregulation relative to the Universal Human Reference RNA (Stratagene). Every horizontal line represents one breast cancer patient, for whom both a tumor and a normal breast sample was available.


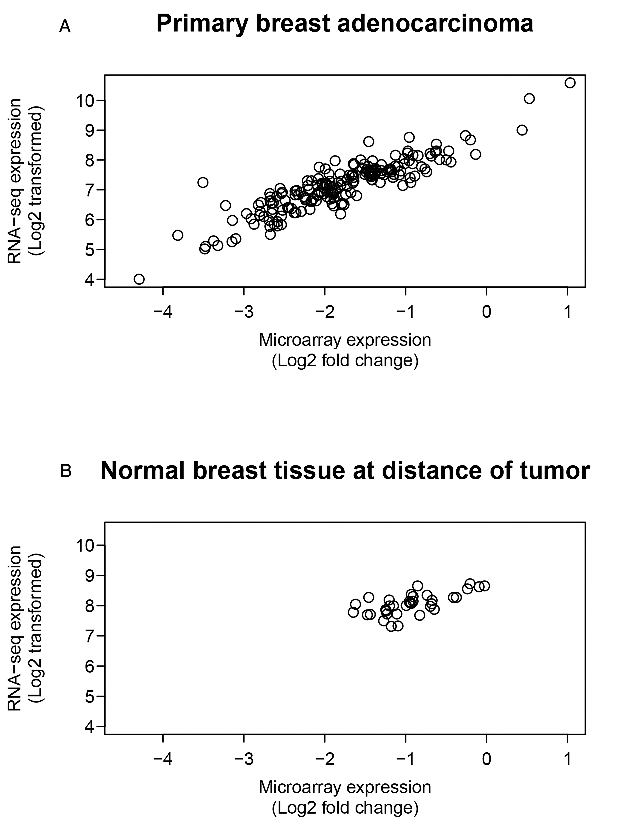


**Figure S2. Correlation between microarray and RNA-seq expression data.** In panel A the correlation between *DFNA5* microarray and RNA-seq expression data is shown for the breast adenocarcinomas. In panel B the same is shown for the normal breast samples. The Spearman correlation coefficient was 0.87 for the tumor samples (panel A), and 0.63 for the normal samples (panel B). The higher correlation in the breast adenocarcinomas compared to the normal samples is, at least in part, attributable to a larger variance in expression levels in the breast adenocarcinomas, both for the microarray and the RNA-seq *DFNA5* expression data. Negative expression values for the microarray data indicate a downregulation relative to the Universal Human Reference RNA (Stratagene).

Table S2. Stepwise linear regression models of *DFNA5* microarray expression on *DFNA5* methylation for both breast adenocarcinoma and normal breast samples.

| **Coefficients** | **Estimate slope** | **S.E.** | **P-value** |
| --- | --- | --- | --- |
| **Breast adenocarcinomas: R² = 0.17 (N = 191)** | | | |
| (Intercept) | -2.45 | 0.27 | 2.0*10^-16^ |
| CpG09333471 | -0.87 | 0.37 | 0.020 |
| CpG19260663 | 1.74 | 0.30 | 2.8*10^-08^ |
| CpG20764575 | -0.55 | 0.31 | 0.079 |
|  | | | |
| **Normal breast samples: R² = 0.44 (N = 36)** | | | |
| (Intercept) | 5.10 | 2.65 | 0.064 |
| CpG07293520 | -19.24 | 11.01 | 0.091 |
| CpG09333471 | 3.87 | 2.16 | 0.083 |
| CpG17569154 | 2.27 | 1.18 | 0.064 |
| CpG17790129 | -4.12 | 2.21 | 0.073 |
| CpG19260663 | -5.32 | 2.65 | 0.054 |
| CpG19706795 | 4.49 | 1.86 | 0.023 |
| CpG20764575 | -4.35 | 2.07 | 0.044 |

Table S3. Stepwise linear regression model of *DFNA5* RNA-seq expression on *DFNA5* methylation for the breast adenocarcinomas.

| **Coefficients** | **Estimate slope** | **S.E.** | **P-value** |
| --- | --- | --- | --- |
| **Breast adenocarcinomas: R² = 0.20 (N = 666)** | | | |
| (Intercept) | 6.02 | 0.37 | < 2.0*10^-16^ |
| CpG04317854 | 0.66 | 0.86 | 0.44 |
| CpG07504598 | -0.63 | 0.70 | 0.37 |
| CpG09333471 | 0.89 | 0.71 | 0.21 |
| CpG19260663 | -1.28 | 0.91 | 0.16 |
| CpG26712096 | 3.83 | 0.81 | 2.78*10^-06^ |
| CpG04317854:CpG07504598 | -3.29 | 1.09 | 2.7*10^-3^ |
| CpG04317854:CpG19260663 | 2.42 | 1.04 | 0.012 |
| CpG07504598:CpG19260663 | 3.19 | 1.12 | 4.6*10^-3^ |
| CpG09333471:CpG26712096 | -2.24 | 0.93 | 0.016 |
| CpG19260663:CpG26712096 | -3.19 | 0.93 | 6.6*10^-4^ |


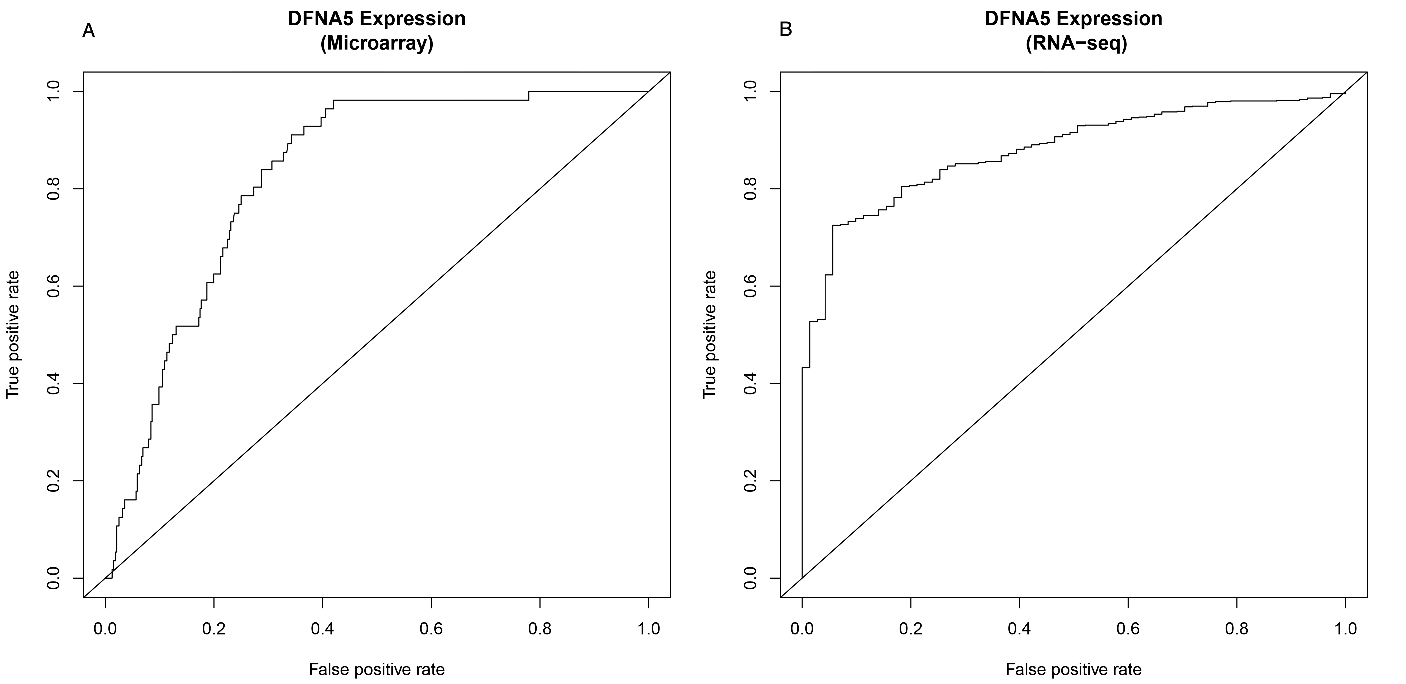


**Figure S3. *DFNA5* expression as biomarker for breast adenocarcinomas.** In panel A the ROC curve for the microarray expression values is shown. The 10-fold cross-validated AUC was 0.82 [95% CI: 0.78 – 0.87] in our dataset. Sensitivities and specificities at the different cutoff values for the predicted probabilities are shown. For example, at a cutoff value of -1.18, a sensitivity of 62.5% and a specificity of 80.0% for detection of breast adenocarcinomas is reached, with an overall accuracy of 78.0% in our dataset. In panel B the ROC curve for the RNA-seq expression values is shown. A ROC curve with a 10-fold cross-validated AUC of 0.88 [95% CI: 0.85 – 0.91] was reached in our dataset. Sensitivities and specificities at the different cutoff values for the predicted probabilities are shown. For example, at a cutoff value of 7.86, a sensitivity of 80.5% and a specificity of 81.7% for detection of breast adenocarcinomas is reached, with an overall accuracy of 80.6% in our dataset. The diagonal line represents the line of no discrimination between breast adenocarcinoma and normal breast samples.

Table S4. Mean *DFNA5* methylation for the ductal and the lobular breast adenocarcinomas for every of the 22 CpGs.

| **CpG** | **Genomic coordinate (GRCh37)** | ***DFNA5* methylation (Ductal)** | | ***DFNA5* methylation (Lobular)** | | **P-value** |
| --- | --- | --- | --- | --- | --- | --- |
|  |  | **Mean** | **95% CI** | **Mean** | **95% CI** |  |
| CpG17790129 | 24738572 | 0.760 | 0.739 - 0.781 | 0.760 | 0.729 - 0.792 | 0.99 |
| CpG14205998 | 24748668 | 0.782 | 0.762 - 0.801 | 0.806 | 0.779 - 0.833 | 0.17 |
| CpG04317854 | 24762562 | 0.738 | 0.721 - 0.756 | 0.764 | 0.738 - 0.790 | 0.13 |
| CpG12922093 | 24767644 | 0.672 | 0.653 - 0.692 | 0.668 | 0.639 - 0.696 | 0.87 |
| CpG17569154 | 24781545 | 0.682 | 0.663 - 0.702 | 0.692 | 0.666 - 0.719 | 0.57 |
| CpG19260663 | 24791121 | 0.739 | 0.721 - 0.758 | 0.750 | 0.726 - 0.774 | 0.57 |
| CpG09333471 | 24796355 | 0.341 | 0.327 - 0.356 | 0.353 | 0.333 - 0.374 | 0.60 |
| CpG00473134* | 24796494 | 0.194 | 0.180 – 0.208 | 0.229 | 0.207 – 0.250 | 0.025 |
| CpG03995857* | 24796553 | 0.269 | 0.250 - 0.289 | 0.331 | 0.303 - 0.360 | 1.3*10^-3^ |
| CpG07320646 | 24796981 | 0.173 | 0.151 - 0.194 | 0.228 | 0.191 - 0.266 | 0.051 |
| CpG07293520* | 24797192 | 0.121 | 0.106 - 0.136 | 0.160 | 0.134 - 0.186 | 0.038 |
| CpG04770504* | 24797363 | 0.167 | 0.151 - 0.184 | 0.229 | 0.202 - 0.256 | 1.5*10^-3^ |
| CpG24805239* | 24797486 | 0.215 | 0.199 - 0.230 | 0.284 | 0.258 - 0.309 | 1.0*10^-4^ |
| CpG01733570 | 24797656 | 0.494 | 0.475 - 0.513 | 0.528 | 0.503 - 0.553 | 0.073 |
| CpG25723149* | 24797680 | 0.468 | 0.449 - 0.487 | 0.535 | 0.509 - 0.562 | 8.0*10^-4^ |
| CpG22804000* | 24797691 | 0.419 | 0.401 - 0.437 | 0.468 | 0.443 - 0.493 | 0.011 |
| CpG07504598* | 24797786 | 0.588 | 0.570 - 0.606 | 0.636 | 0.614 - 0.657 | 5.2*10^-3^ |
| CpG15037663 | 24797835 | 0.596 | 0.577 - 0.614 | 0.629 | 0.606 - 0.652 | 0.051 |
| CpG19706795* | 24797839 | 0.656 | 0.639 - 0.673 | 0.698 | 0.677 - 0.718 | 0.013 |
| CpG20764575 | 24797884 | 0.651 | 0.634 - 0.669 | 0.674 | 0.655 - 0.694 | 0.091 |
| CpG06301139* | 24798175 | 0.829 | 0.815 - 0.844 | 0.863 | 0.845 - 0.880 | 0.011 |
| CpG26712096 | 24798855 | 0.725 | 0.705 - 0.745 | 0.747 | 0.718 - 0.775 | 0.19 |

The mean *DFNA5* methylation with 95% CI were reported for every of the 22 CpGs for both the ductal (N = 496) and the lobular (N = 172) adenocarcinomas. The CpGs with a significant p-value for a difference in means between ductal and lobular adenocarcinomas are marked with an asterisk. The CpGs in dark grey are located in the gene body, the CpGs in intermediate grey are located in the putative gene promoter and the last two CpGs, indicated in light grey, are located upstream from the putative gene promoter.

Table S5. Mean *DFNA5* methylation for ER status, PR status and *HER2* status for every of the 22 CpGs.

|  |  | **ER +** *(N = 490)* | | **ER –** *(N = 142)* | | **PR +** *(N = 428)* | | **PR –** *(N = 201)* | | ***HER2*+** *(N = 32)* | | ***HER2*–** *(N = 195)* | |
| --- | --- | --- | --- | --- | --- | --- | --- | --- | --- | --- | --- | --- | --- |
| **CpG** | **Genomic coordinate (GRCh37)** | **Mean** | **95% CI** | **Mean** | **95% CI** | **Mean** | **95% CI** | **Mean** | **95% CI** | **Mean** | **95% CI** | **Mean** | **95% CI** |
| CpG17790129 | 24738572 | 0.729* | 0.707 - 0.750 | 0.873* | 0.850 - 0.895 | 0.728* | 0.705 - 0.750 | 0.830* | 0.803 - 0.856 | 0.720 | 0.633 - 0.807 | 0.779 | 0.747 - 0.811 |
| CpG14205998 | 24748668 | 0.763* | 0.743 - 0.783 | 0.880* | 0.861 - 0.899 | 0.765* | 0.744 - 0.786 | 0.839* | 0.814 - 0.864 | 0.753 | 0.672 - 0.833 | 0.800 | 0.772 - 0.829 |
| CpG04317854 | 24762562 | 0.727* | 0.710 - 0.745 | 0.804* | 0.782 - 0.826 | 0.728* | 0.709 - 0.747 | 0.778* | 0.754 - 0.802 | 0.667* | 0.595 - 0.740 | 0.752* | 0.726 - 0.778 |
| CpG12922093 | 24767644 | 0.639* | 0.620 - 0.658 | 0.783* | 0.759 - 0.807 | 0.636* | 0.615 - 0.656 | 0.745* | 0.719 - 0.772 | 0.634 | 0.558 - 0.711 | 0.685 | 0.655 - 0.715 |
| CpG17569154 | 24781545 | 0.643* | 0.625 - 0.661 | 0.832* | 0.807 - 0.857 | 0.641* | 0.622 - 0.660 | 0.776* | 0.749 - 0.804 | 0.634 | 0.558 - 0.710 | 0.695 | 0.666 - 0.723 |
| CpG19260663 | 24791121 | 0.709* | 0.691 - 0.727 | 0.853* | 0.831 - 0.874 | 0.706* | 0.687 - 0.725 | 0.814* | 0.791 - 0.837 | 0.749 | 0.678 - 0.819 | 0.755 | 0.728 - 0.782 |
| CpG09333471 | 24796355 | 0.353* | 0.338 - 0.367 | 0.306* | 0.285 - 0.328 | 0.346 | 0.331 - 0.361 | 0.335 | 0.314 - 0.357 | 0.364 | 0.301 - 0.427 | 0.345 | 0.323 - 0.366 |
| CpG00473134 | 24796494 | 0.221* | 0.208 - 0.235 | 0.134* | 0.114 - 0.153 | 0.216* | 0.202 - 0.230 | 0.171* | 0.151 - 0.191 | 0.230 | 0.169 - 0.291 | 0.200 | 0.179 - 0.221 |
| CpG03995857 | 24796553 | 0.316* | 0.297 - 0.334 | 0.164* | 0.134 - 0.194 | 0.307* | 0.288 - 0.326 | 0.230* | 0.200 - 0.261 | 0.360 | 0.273 - 0.447 | 0.289 | 0.258 - 0.320 |
| CpG07320646 | 24796981 | 0.213* | 0.191 - 0.235 | 0.0927* | 0.0623 - 0.123 | 0.198 | 0.175 - 0.221 | 0.164 | 0.130 - 0.198 | 0.271 | 0.173 - 0.369 | 0.188 | 0.152 - 0.223 |
| CpG07293520 | 24797192 | 0.147* | 0.131 - 0.162 | 0.0757* | 0.0534 - 0.0980 | 0.137 | 0.121 - 0.153 | 0.118 | 0.0944 - 0.141 | 0.191 | 0.122 - 0.259 | 0.129 | 0.105 - 0.153 |
| CpG04770504 | 24797363 | 0.212* | 0.196 - 0.228 | 0.0836* | 0.0602 - 0.107 | 0.203* | 0.186 - 0.220 | 0.142* | 0.116 - 0.168 | 0.246 | 0.176 - 0.317 | 0.180 | 0.155 - 0.206 |
| CpG24805239 | 24797486 | 0.257* | 0.242 - 0.273 | 0.142* | 0.118 - 0.166 | 0.251* | 0.235 - 0.268 | 0.190* | 0.165 - 0.215 | 0.288 | 0.212 - 0.365 | 0.232 | 0.207 - 0.257 |
| CpG01733570 | 24797656 | 0.512* | 0.494 - 0.530 | 0.471* | 0.438 - 0.504 | 0.509 | 0.490 - 0.528 | 0.491 | 0.462 - 0.520 | 0.503 | 0.439 - 0.567 | 0.524 | 0.496 - 0.552 |
| CpG25723149 | 24797680 | 0.511* | 0.493 - 0.529 | 0.388* | 0.357 - 0.418 | 0.508* | 0.489 - 0.526 | 0.433* | 0.403 - 0.462 | 0.477 | 0.400 - 0.553 | 0.504 | 0.475 - 0.532 |
| CpG22804000 | 24797691 | 0.450* | 0.433 - 0.467 | 0.367* | 0.337 - 0.396 | 0.448* | 0.430 - 0.465 | 0.399* | 0.372 - 0.426 | 0.428 | 0.363 - 0.493 | 0.448 | 0.421 - 0.475 |
| CpG07504598 | 24797786 | 0.622* | 0.607 - 0.638 | 0.524* | 0.490 - 0.558 | 0.622* | 0.605 - 0.638 | 0.557* | 0.529 - 0.586 | 0.581 | 0.519 - 0.643 | 0.627 | 0.601 - 0.653 |
| CpG15037663 | 24797835 | 0.612 | 0.594 - 0.629 | 0.580 | 0.546 - 0.614 | 0.609 | 0.590 - 0.627 | 0.597 | 0.569 - 0.625 | 0.592 | 0.523 - 0.661 | 0.625 | 0.598 - 0.652 |
| CpG19706795 | 24797839 | 0.673 | 0.657 - 0.688 | 0.645 | 0.614 - 0.677 | 0.671 | 0.654 - 0.687 | 0.660 | 0.634 - 0.686 | 0.648 | 0.584 - 0.711 | 0.686 | 0.661 - 0.711 |
| CpG20764575 | 24797884 | 0.674* | 0.660 - 0.688 | 0.599* | 0.563 - 0.634 | 0.673* | 0.658 - 0.688 | 0.626* | 0.597 - 0.654 | 0.639 | 0.571 - 0.706 | 0.672 | 0.647 - 0.697 |
| CpG06301139 | 24798175 | 0.830* | 0.816 - 0.845 | 0.858* | 0.838 - 0.878 | 0.831 | 0.815 - 0.846 | 0.850 | 0.831 - 0.869 | 0.817 | 0.764 - 0.871 | 0.846 | 0.826 - 0.867 |
| CpG26712096 | 24798855 | 0.697* | 0.677 - 0.717 | 0.851* | 0.826 - 0.876 | 0.696* | 0.675 - 0.716 | 0.807* | 0.780 - 0.834 | 0.710 | 0.632 - 0.787 | 0.752 | 0.722 - 0.781 |

The mean *DFNA5* methylation with 95% CI were reported for every of the 22 CpGs for ER status, PR status and *HER2* status. The significant differences in mean *DFNA5* methylation between two categories are marked with an asterisk. The CpGs in dark grey are located in the gene body, the CpGs in intermediate grey are located in the putative gene promoter, and the last two CpGs, indicated in light grey, are located upstream from the putative gene promoter.

Table S6. Mean *DFNA5* expression for ER+ and ER- breast adenocarcinomas.

| ***DFNA5* microarray expression (log2 fc)** | | | ***DFNA5* RNA-seq expression (log2)** | | |
| --- | --- | --- | --- | --- | --- |
|  | **Mean** | **95% CI** |  | **Mean** | **95% CI** |
| **ER+ breast adenocarcinomas** (N = 362) | -1.87 | -1.95 – -1.79 | **ER+ breast adenocarcinomas** (N = 488) | 7.17 | 7.10 – 7.23 |
| **ER- breast adenocarcinomas** (N = 107) | -1.68 | -1.85 – -1.50 | **ER- breast adenocarcinomas** (N = 142) | 7.39 | 7.24 – 7.55 |

Table S7. Mean *DFNA5* methylation for the four tumor stages for every of the 22 CpGs.

|  |  | **Stage I** *(N = 109)* | | **Stage II** *(N = 378)* | | **Stage III** *(N = 166)* | | **Stage IV** *(N = 9)* | |
| --- | --- | --- | --- | --- | --- | --- | --- | --- | --- |
| **CpG** | **Genomic coordinate (GRCh37)** | **Mean** | **95% CI** | **Mean** | **95% CI** | **Mean** | **95% CI** | **Mean** | **95% CI** |
| CpG17790129 | 24738572 | 0.805 | 0.769 - 0.841 | 0.759 | 0.735 - 0.783 | 0.731 | 0.693 - 0.768 | 0.829 | 0.710 - 0.948 |
| CpG14205998 | 24748668 | 0.814 | 0.778 - 0.850 | 0.787 | 0.766 - 0.808 | 0.773 | 0.739 - 0.807 | 0.835 | 0.740 - 0.930 |
| CpG04317854 | 24762562 | 0.773 | 0.741 - 0.804 | 0.738 | 0.719 - 0.758 | 0.739 | 0.708 - 0.769 | 0.793 | 0.679 - 0.906 |
| CpG12922093 | 24767644 | 0.691 | 0.653 - 0.730 | 0.677 | 0.655 - 0.698 | 0.641 | 0.608 - 0.675 | 0.697 | 0.549 - 0.845 |
| CpG17569154 | 24781545 | 0.689 | 0.649 - 0.728 | 0.695 | 0.674 - 0.716 | 0.656 | 0.625 - 0.688 | 0.690 | 0.549 - 0.832 |
| CpG19260663 | 24791121 | 0.759 | 0.722 - 0.795 | 0.745 | 0.725 - 0.764 | 0.725 | 0.694 - 0.756 | 0.724 | 0.570 - 0.878 |
| CpG09333471 | 24796355 | 0.347 | 0.320 - 0.375 | 0.343 | 0.326 - 0.360 | 0.344 | 0.321 - 0.367 | 0.351 | 0.270 - 0.432 |
| CpG00473134 | 24796494 | 0.211 | 0.184 - 0.238 | 0.195 | 0.180 - 0.211 | 0.220 | 0.196 - 0.243 | 0.144 | 0.0638 - 0.225 |
| CpG03995857* | 24796553 | 0.278 | 0.237 - 0.320 | 0.269 | 0.248 - 0.290 | 0.324 | 0.292 - 0.356 | 0.323 | 0.158 - 0.489 |
| CpG07320646* | 24796981 | 0.161 | 0.117 - 0.205 | 0.166 | 0.143 - 0.189 | 0.252 | 0.210 - 0.294 | 0.194 | -0.0134 - 0.401 |
| CpG07293520* | 24797192 | 0.113 | 0.0834 - 0.143 | 0.117 | 0.101 - 0.132 | 0.174 | 0.144 - 0.204 | 0.134 | -0.0017 - 0.269 |
| CpG04770504* | 24797363 | 0.157 | 0.123 - 0.191 | 0.171 | 0.153 - 0.189 | 0.229 | 0.200 - 0.258 | 0.159 | 0.0354 - 0.282 |
| CpG24805239* | 24797486 | 0.218 | 0.186 - 0.250 | 0.222 | 0.204 0.240 | 0.269 | 0.241 - 0.296 | 0.216 | 0.0889 - 0.344 |
| CpG01733570 | 24797656 | 0.518 | 0.483 - 0.554 | 0.498 | 0.477 - 0.518 | 0.500 | 0.466 - 0.535 | 0.521 | 0.415 - 0.626 |
| CpG25723149 | 24797680 | 0.491 | 0.456 - 0.526 | 0.476 | 0.454 - 0.497 | 0.497 | 0.464 - 0.530 | 0.521 | 0.419 - 0.623 |
| CpG22804000 | 24797691 | 0.443 | 0.409 - 0.476 | 0.429 | 0.409 - 0.449 | 0.426 | 0.396 - 0.455 | 0.470 | 0.368 - 0.571 |
| CpG07504598 | 24797786 | 0.619 | 0.590 - 0.648 | 0.596 | 0.577 - 0.616 | 0.589 | 0.559 - 0.619 | 0.655 | 0.559 - 0.752 |
| CpG15037663 | 24797835 | 0.631 | 0.599 - 0.663 | 0.605 | 0.584 - 0.625 | 0.581 | 0.550 - 0.611 | 0.660 | 0.556 - 0.764 |
| CpG19706795 | 24797839 | 0.698 | 0.671 - 0.724 | 0.672 | 0.653 - 0.691 | 0.633 | 0.604 - 0.662 | 0.674 | 0.588 - 0.761 |
| CpG20764575 | 24797884 | 0.669 | 0.645 - 0.694 | 0.658 | 0.638 - 0.677 | 0.645 | 0.617 - 0.673 | 0.662 | 0.566 - 0.758 |
| CpG06301139 | 24798175 | 0.865 | 0.842 - 0.889 | 0.835 | 0.819 - 0.851 | 0.824 | 0.799 - 0.849 | 0.840 | 0.764 - 0.916 |
| CpG26712096 | 24798855 | 0.738 | 0.699 - 0.778 | 0.737 | 0.716 - 0.759 | 0.711 | 0.676 - 0.745 | 0.718 | 0.576 - 0.860 |

The mean *DFNA5* methylation with 95% CI were reported for every of the 22 CpGs for every of the four tumor stages. The CpGs with a significant p-value for a difference in mean *DFNA5* methylation between the four categories are indicated with an asterisk. Remarkably, those five CpGs are located, next to each other, in the gene promoter region. The highest *DFNA5* promotor methylation was seen in stage III. Stage I and II showed an almost identical *DFNA5* promotor methylation. The CpGs in dark grey are located in the gene body, the CpGs in intermediate grey are located in the putative gene promoter, and the last two CpGs, indicated in light grey, are located upstream from the putative gene promoter.

**Table S8. Vital status of the breast adenocarcinoma patients after 5 years follow-up.**

|  | **Alive** | **Dead** | **Total** |
| --- | --- | --- | --- |
| ***DFNA5* methylation** |  |  |  |
| All breast adenocarcinomas | 549 | 24 | 573 |
| Ductal adenocarcinomas | 403 | 21 | 424 |
| Lobular adenocarcinomas | 146 | 3 | 149 |
| ***DFNA5* microarray expression** |  |  |  |
| All breast adenocarcinomas | 365 | 15 | 380 |
| Ductal adenocarcinomas | 335 | 15 | 350 |
| Lobular adenocarcinomas | 30 | 0 | 30 |
| ***DFNA5* RNA-seq expression** |  |  |  |
| All breast adenocarcinomas | 548 | 24 | 572 |
| Ductal adenocarcinomas | 402 | 21 | 423 |
| Lobular adenocarcinomas | 146 | 3 | 149 |

The number of patients in each category are reported for the *DFNA5* methylation and expression (both microarray and RNA-seq) dataset. Only cases with no missing values of any of the covariates were included.

Table S9. False Discovery Rate (FDR) for 5-year OS analysis on all breast adenocarcinomas and ductal breast adenocarcinomas.

| **All breast adenocarcinoma** | | |  | **Ductal breast adenocarcinoma** | | |
| --- | --- | --- | --- | --- | --- | --- |
| **CpG** | **P-value** | **Q-value** |  | **CpG** | **P-value** | **Q-value** |
| CpG19260663 | 4.20*10^-3^ * | 0.067 |  | CpG19260663 | 5.5*10^-3^ * | 0.046 |
| CpG17790129 | 8.2*10^-3^ * | 0.067 |  | CpG12922093 | 6.2*10^-3^ * | 0.046 |
| CpG12922093 | 0.012 * | 0.067 |  | CpG17569154 | 6.3*10^-3^ * | 0.046 |
| CpG17569154 | 0.012 * | 0.067 |  | CpG17790129 | 0.019* | 0.099 |
| CpG14205998 | 0.015 * | 0.067 |  | CpG14205998 | 0.023* | 0.099 |
| CpG26712096 | 0.053 | 0.18 |  | CpG26712096 | 0.036* | 0.13 |
| CpG09333471 | 0.059 | 0.18 |  | CpG06301139 | 0.052 | 0.16 |
| CpG06301139 | 0.064 | 0.18 |  | CpG04317854 | 0.064 | 0.18 |
| CpG04317854 | 0.16 | 0.38 |  | CpG04770504 | 0.098 | 0.24 |
| CpG04770504 | 0.27 | 0.60 |  | CpG07320646 | 0.22 | 0.44 |
| CpG01733570 | 0.34 | 0.68 |  | CpG09333471 | 0.22 | 0.44 |
| CpG20764575 | 0.46 | 0.85 |  | CpG07293520 | 0.29 | 0.53 |
| CpG07320646 | 0.55 | 0.90 |  | CpG01733570 | 0.44 | 0.73 |
| CpG00473134 | 0.57 | 0.90 |  | CpG03995857 | 0.49 | 0.73 |
| CpG07504598 | 0.77 | 0.93 |  | CpG24805239 | 0.49 | 0.73 |
| CpG19706795 | 0.80 | 0.93 |  | CpG20764575 | 0.57 | 0.76 |
| CpG15037663 | 0.85 | 0.93 |  | CpG19706795 | 0.58 | 0.76 |
| CpG03995857 | 0.85 | 0.93 |  | CpG00473134 | 0.62 | 0.76 |
| CpG24805239 | 0.87 | 0.93 |  | CpG25723149 | 0.86 | 0.94 |
| CpG25723149 | 0.89 | 0.93 |  | CpG07504598 | 0.91 | 0.94 |
| CpG07293520 | 0.89 | 0.93 |  | CpG22804000 | 0.94 | 0.94 |
| CpG22804000 | 0.93 | 0.93 |  | CpG15037663 | 0.94 | 0.94 |

The CpGs are sorted by their p-values. Q-values indicate the expected fraction of false positive findings, in case the p-value with a given q-value is called significant [1].

1. Benjamini Y, Hochberg Y. Controlling the false discovery rate: a practical and powerful approach to multiple testing. JSTOR. 1995.

**Table S10. Concordance for 5-year OS analysis on all breast adenocarcinomas.**

| **Cox proportional hazard model with age, separated by stage = Basic Model** | | | | |
| --- | --- | --- | --- | --- |
| **Stage** | **Concordant** | **Discordant** | **Concordance** |  |
| **1** | 21 | 5 | 0.796 |  |
| **2** | 1029 | 464 | 0.685 |  |
| **3** | 629 | 320 | 0.659 |  |
| **4** | 5 | 6 | 0.455 |  |
|  |  |  |  |  |
| **Cox proportional hazard model with age and *DFNA5* CpG methylation, separated by stage** | | | | |
| **CpG12922093** |  |  |  |  |
| **Stage** | **Concordant** | **Discordant** | **Concordance** | **Difference in concordance with basic model** |
| **1** | 24 | 3 | 0.889 | 0.0926 |
| **2** | 1173 | 352 | 0.769 | 0.0839 |
| **3** | 687 | 283 | 0.708 | 0.0490 |
| **4** | 9 | 2 | 0.818 | 0.364 |
|  |  |  |  |  |
| **CpG14205998** |  |  |  |  |
| **Stage** | **Concordant** | **Discordant** | **Concordance** | **Difference in concordance with basic model** |
| **1** | 25 | 2 | 0.926 | 0.130 |
| **2** | 1194 | 331 | 0.783 | 0.0977 |
| **3** | 715 | 255 | 0.737 | 0.0778 |
| **4** | 8 | 3 | 0.727 | 0.273 |
|  |  |  |  |  |
| **CpG17569154** |  |  |  |  |
| **Stage** | **Concordant** | **Discordant** | **Concordance** | **Difference in concordance with basic model** |
| **1** | 23 | 4 | 0.852 | 0.0556 |
| **2** | 1152 | 373 | 0.755 | 0.0702 |
| **3** | 692 | 278 | 0.713 | 0.0541 |
| **4** | 9 | 2 | 0.818 | 0.364 |
|  |  |  |  |  |
| **CpG17790129** |  |  |  |  |
| **Stage** | **Concordant** | **Discordant** | **Concordance** | **Difference in concordance with basic model** |
| **1** | 23 | 4 | 0.852 | 0.0556 |
| **2** | 1195 | 330 | 0.784 | 0.0984 |
| **3** | 746 | 224 | 0.769 | 0.110 |
| **4** | 9 | 2 | 0.818 | 0.364 |
|  |  |  |  |  |
| **CpG19260663** |  |  |  |  |
| **Stage** | **Concordant** | **Discordant** | **Concordance** | **Difference in concordance with basic model** |
| **1** | 21 | 6 | 0.778 | -0.0185 |
| **2** | 1214 | 311 | 0.796 | 0.111 |
| **3** | 712 | 258 | 0.734 | 0.0747 |
| **4** | 9 | 2 | 0.818 | 0.364 |

The basic model is reported in the upper table, where 5-year OS time is determined only on basis of the four tumor stages and age. Subsequently, five models (for each of the five significant CpGs) were made were 5-year OS time is determined on basis of the four tumor stages, age and each time one of the significant CpGs. The column “Difference in concordance with basic model” shows the difference in concordance between each of these five models and the basic model.

Table S11. Concordance for 5-year OS analysis on ductal breast adenocarcinomas.

| **Cox proportional hazard model with age, separated by stage = Basic Model** | | | | |
| --- | --- | --- | --- | --- |
| **Stage** | **Concordant** | **Discordant** | **Concordance** |  |
| **1** | 18 | 2 | 0.900 |  |
| **2** | 690 | 330 | 0.673 |  |
| **3** | 340 | 192 | 0.636 |  |
| **4** | 5 | 6 | 0.455 |  |
|  |  |  |  |  |
| **Cox proportional hazard model with age and *DFNA5* CpG methylation, separated by stage** | | | | |
| **CpG12922093** |  |  |  |  |
| **Stage** | **Concordant** | **Discordant** | **Concordance** | **Difference in concordance with basic model** |
| **1** | 18 | 2 | 0.900 | 0 |
| **2** | 759 | 281 | 0.730 | 0.0567 |
| **3** | 395 | 148 | 0.727 | 0.0912 |
| **4** | 9 | 2 | 0.818 | 0.364 |
|  |  |  |  |  |
| **CpG14205998** |  |  |  |  |
| **Stage** | **Concordant** | **Discordant** | **Concordance** | **Difference in concordance with basic model** |
| **1** | 18 | 2 | 0.900 | 0 |
| **2** | 812 | 228 | 0.781 | 0.108 |
| **3** | 394 | 149 | 0.726 | 0.0893 |
| **4** | 8 | 3 | 0.727 | 0.273 |
|  |  |  |  |  |
| **CpG17569154** |  |  |  |  |
| **Stage** | **Concordant** | **Discordant** | **Concordance** | **Difference in concordance with basic model** |
| **1** | 18 | 2 | 0.900 | 0 |
| **2** | 765 | 275 | 0.736 | 0.0625 |
| **3** | 397 | 146 | 0.731 | 0.0948 |
| **4** | 9 | 2 | 0.818 | 0.364 |
|  |  |  |  |  |
| **CpG17790129** |  |  |  |  |
| **Stage** | **Concordant** | **Discordant** | **Concordance** | **Difference in concordance with basic model** |
| **1** | 18 | 2 | 0.900 | 0 |
| **2** | 807 | 233 | 0.776 | 0.103 |
| **3** | 424 | 119 | 0.781 | 0.145 |
| **4** | 9 | 2 | 0.818 | 0.364 |
|  |  |  |  |  |
| **CpG19260663** |  |  |  |  |
| **Stage** | **Concordant** | **Discordant** | **Concordance** | **Difference in concordance with basic model** |
| **1** | 14 | 6 | 0.700 | -0.200 |
| **2** | 796 | 244 | 0.765 | 0.0923 |
| **3** | 404 | 139 | 0.744 | 0.108 |
| **4** | 9 | 2 | 0.818 | 0.364 |

| **CpG26712096** |  |  |  |  |
| --- | --- | --- | --- | --- |
| **Stage** | **Concordant** | **Discordant** | **Concordance** | **Difference in concordance with basic model** |
| **1** | 11 | 9 | 0.550 | -0.350 |
| **2** | 745 | 295 | 0.716 | 0.0433 |
| **3** | 403 | 140 | 0.742 | 0.106 |
| **4** | 7 | 4 | 0.636 | 0.182 |

The basic model is reported in the upper table, where 5-year OS time is determined only on basis of the four tumor stages and age. Subsequently, six models (for each of the six significant CpGs) were made were 5-year OS time is determined on basis of the four tumor stages, age and each time one of the significant CpGs. The column “Difference in concordance with basic model” shows the difference in concordance between each of these six models and the basic model.

**Table S12. Similarities and differences between three studies investigating *DFNA5* methylation in breast cancer.**

|  | **Kim et al.** | **Croes et al. – clinical samples** | **Croes et al. – TCGA** |
| --- | --- | --- | --- |
| **Year of publication** | 2008 | 2017 | 2018 |
| **Number of samples**  Breast adenocarcinomas  Normal breast tissue at distance of tumor  Healthy breast reductions | 34  13  7 | 123  16  24 | 668  85  0 |
| **Technology** | TaqMan-MSP | Pyrosequencing | Infinium HumanMethylation 450k |
| **CpGs analyzed** | See figure 2 | See figure 2 | See figure 2 |
| **Association *DFNA5* methylation with**  Tumor/Normal  Tumor subtype (ductal/lobular)  ER state  PgR state  *HER2* state  Stage (I-IV)  Tumor Grade  MAI  Tumor Diameter  Vascular Invasion  Lymphatic Invasion  Lymph Node Metastasis  5-year Overall Survival | Yes  N.A.  No (n = 24)  No (n = 24)  N.A.  No (n = 26)  No (n = 24)  N.A.  No (n = 26)  No (n = 23)  No (n = 23)  Yes (n = 21)  N.A. | Yes  N.A.  No (n = 119)  No (n = 114)  Yes (n = 58)  No (n = 97)  No (n = 101)  No ( n = 80)  No (n = 102)  N.A.  No (n = 49)  N.A.  No (n = 120) | Yes  Yes (n = 668)  Yes (20/22 CpGs; n = 632)  Yes (15/22 CpGs; n = 629)  Yes (1/22 CpGs; n = 227)  Yes (5/22 CpGs; n = 662)  N.A.  N.A.  N.A.  N.A.  N.A.  N.A.  Yes (5/22 CpGs; n = 573) |
| **Statistics** | Chi-square | Linear regression  Kaplan meier  Cox proportional hazard | Paired samples *t*-test  Linear mixed model  Cox proportional hazard |

To date there are three studies that investigated *DFNA5* methylation in clinical breast cancer samples (Kim et al., 2008; Croes et al., 2017 and the current study). These studies are different in respect to the techniques used, the CpGs analyzed, the number of samples (per parameter), the statistics used and consequently the associations found. The numbers of adenocarcinomas tested for the association with *DFNA5* methylation are reported between brackets for each parameter.

**Table S13. Single nucleotide variants in the *DFNA5* gene with corresponding changes in the amino acid sequence of DFNA5.**

| **Patient** | **Age at diagnosis (years)** | **Histological diagnosis** | **Tumor stage** | **ER** | **PR** | ***HER2*** | **Variation classification** | **Protein change** | **Prediction** | **SIFT score** |
| --- | --- | --- | --- | --- | --- | --- | --- | --- | --- | --- |
| 1 | 39 | ductal | II | ER+ | PR+ | *HER2-* | Silent | p.G369G | Tolerated | 1 |
| 2 | 45 | ductal | II | ER- | PR- | *HER2-* | Missense | p.E347G | Damaging | 0.05 |
| 3 | 64 | lobular | III | ER+ | PR+ | *HER2-* | Missense | p.T57P | Damaging | 0 |
| 4 | 60 | ductal | / | ER- | PR- | / | Silent | p.V226V | Tolerated | 1 |
| 5 | 54 | ductal | II | ER+ | PR+ | / | Missense | p.D267H | Tolerated | 0.38 |

*DFNA5* sequencing data (level 2) were obtained from TCGA whole exome sequencing (WES) experiments on an Illumina HiSeq platform (Illumina Inc., San Diego, California, USA). Somatic mutations (coding, splice site and validated non-coding somatic variants) were available in Mutation Annotation Format (MAF, v2.4.1). We used the SIFT Human Protein predction program, which provides SIFT predictions for all Ensembl transcripts with an assigned ENSP number and returns tolerated/intolerated mutations for selected amino acid substitutions. The SIFT score ranges from 0 to 1. The amino acid substitution is predicted damaging if the score is <= 0.05, and tolerated if the score is > 0.05. *DFNA5* WES data were available for 570 primary breast adenocarcinoma samples. In five patients, a somatic single nucleotide variation (SNV) was identified in the *DFNA5* gene. Three of these SNVs are missense and two are silent.

Table S14. Three methylation datasets from the Gene Expression Omnibus (GEO) for validation of our model to predict the tumor status.

| **GEO accession number** | **Number of breast cancers** | **Number of normal breast tissues at a distance of the tumor** | **Number of normal breast tissues from healthy women** |
| --- | --- | --- | --- |
| GSE52865 | 40 | 17 | / |
| GSE69914 | 305 | 42 | 50 |
| GSE60185 | 186 | / | 46 |
